# Factors Affecting Solvent Retention due to Gel Formation during Dissolution-Based Plastic Recycling

**DOI:** 10.1021/acssuschemeng.5c13798

**Published:** 2026-05-02

**Authors:** Hedam Kim, Ali Altamimi, Whitney S. Loo, Reid C. Van Lehn, George W. Huber

**Affiliations:** † Department of Chemical and Biological Engineering, 5228 University of Wisconsin-Madison, Madison, Wisconsin 53706, United States; ‡ Department of Chemistry, University of Wisconsin-Madison, Madison, Wisconsin 53706, United States

**Keywords:** solvent retention, solvent removal, dissolution-based recycling, STRAP, plastic recycling, machine learning, support vector regression

## Abstract

Solvent-targeted recovery and precipitation (STRAP) is a dissolution-based plastic recycling process that allows the selective recovery of polymers from mixed plastic waste, but removing the residual solvent from polymer gels remains a major challenge. In this study, we investigate factors affecting the residual solvent in gels during dissolution-based recycling. Experimental measurements show that the precipitation method, cooling rate, and extended filtration time do not have a significant effect on solvent retention. We investigated six variables (solvent vapor pressure, polymer molecular weight, initial polymer-to-solvent mass ratio, predicted solubility, the total Hansen solubility parameter, and the Flory–Huggins interaction parameter) that influence solvent retention. The six variables were used to build a support vector regression-based model to predict solvent retention and determine feature contribution for six polymers commonly used in recycling and nine solvents. Our analysis reveals that solvent vapor pressure is the most influential factor, followed by polymer molecular weight, while the Flory–Huggins interaction parameter has a weaker influence. The predictive framework established in this work provides a foundation for selecting polymer–solvent systems that minimize solvent retention in dissolution-based plastic recycling.

## Graphical abstract

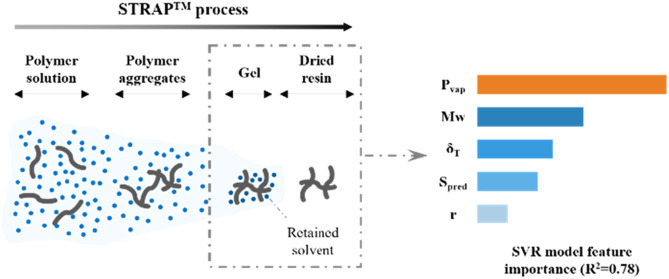

## 1 Introduction

Due to their low cost and wide versatility, plastics are one of the most widely produced materials with a current annual production of 380 million tons and a cumulative amount expected to reach 30 billion tons by 2050.^1,2^ This massive production has led to environmental challenges and drawn attention to the challenges with the end-of-life usage of plastics.^3^ Solvent-targeted recovery and precipitation (STRAP), first proposed by Walker et al., leverages polymer solubility differences to selectively recover polymers from multilayer plastic films.^4^ STRAP offers several advantages, including impurity removal, deep cleaning of polymer solutions, and no degradation even after multiple recycling cycles.^5^ The process consists of four main steps: targeted polymer dissolution, separation of the dissolved fraction, precipitation to recover a targeted polymer, and subsequent vacuum filtration and drying to remove the retained solvent.^5–10^

In our prior work, Zhou et al. established a computational approach to select solvents for STRAP that lead to high polymer solubility at an elevated temperature (*e*.*g*., 90–110 °C) and near-zero solubility at room temperature, permitting initial high-temperature selective dissolution followed by cooling to precipitate the dissolved polymer.^11^ During dissolution at elevated temperature, polymer chains dissolve homogeneously in the solvent (Figure 1).^12^ Upon cooling, the mixture undergoes phase separation into solvent-rich and polymer-rich domains.^13,14^ In contrast to conventional nonsolvent precipitation, in which polymer solubility is reduced in a mixture of the solvent and a miscible nonsolvent (equivalently referred to as an antisolvent), precipitation in STRAP is induced by cooling and the accompanying loss of polymer solubility. This difference is important because thermally triggered precipitation is governed by heat transfer, which is substantially faster than mass transfer in nonsolvent-based precipitation.^15^ In STRAP, this can favor the formation of a bulk swollen polymer, whereas nonsolvent precipitation may lead to promoted demixing and finer particulate or dispersed aggregate morphologies depending on process conditions.^16^ Vacuum filtration removes the loose or residual solvent and results in a swollen polymer gel. At this stage, Yan et al. reported that 70 wt % of dodecane (an example STRAP solvent) was retained in polyethylene (PE) gels after vacuum filtration.^17^ The solvent persists in the gel because rapid phase separation traps solvent molecules in polymer-rich aggregates.^13^ High-molecular weight polymers are expected to trap more solvent because of restricted solvent diffusion through the polymer matrix.^18^

**Figure 1:**
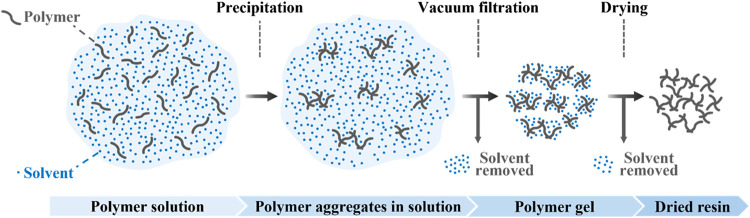
Conceptual schematic of the polymer–solvent system during STRAP. Blue dots and gray lines indicate solvent molecules and polymer chains, respectively.

After vacuum filtration, the solvent is removed thermally in a vacuum oven to form a dried resin that has parts per million levels of solvent (Figure 1). Prior studies have largely emphasized the selective dissolution of different feedstocks, qualities of resulting films, and energy consumption.^6,8–10,17,19^ However, no prior publication has described solvent retention in polymer gels during dissolution-based plastic recycling, to the best of our knowledge. Removing the solvent mechanically is less energy-intensive than removing the solvent by drying. It is, therefore, critical to understand how to reduce the solvent in polymer gels. Our process model by Tushar et al. showed that the dryers in STRAP consumed approximately 7.7 × 10^5^ kJ hr^–1^, corresponding to 25% of the total annual energy use of a STRAP process.^10^ Energy is therefore the most costly and environmentally impactful utility in the process, highlighting the need to maximize mechanical solvent removal (e.g., vacuum filtration), while minimizing thermal drying requirements (e.g., drying).^20^ Furthermore, Yan et al. demonstrated that nonvolatile color bodies remain in the solvent and subsequently stay with the polymer during solvent evaporation, resulting in a colored recycled polymer.^17^ Thus, investigating solvent retention in gels is pivotal not only for reducing energy consumption but also for improving the quality of recycled plastics.

The objective of this work is to understand what factors impact solvent retention in polymer gels formed during STRAP and develop a quantitative model that predicts solvent retention behavior based on the physical properties of the solvent and polymer commonly recycled from plastic waste. In this work, the term “gel” refers to a highly viscous solvent-swollen polymer formed after precipitation and filtration that exhibits no steady-state flow.^21^ In this state, the solvent is physically trapped within the polymer aggregates. As we show in this paper, solvent retention in polymer gels is governed by multiple interacting factors rather than a single physical property. In this study, six variables were considered: (i) polymer–solvent interaction parameters (Flory–Huggins interaction parameter and predicted solubility), (ii) solvent and polymer physical properties (solvent vapor pressure, solvent total Hansen solubility parameter, and polymer molecular weight), and (iii) polymer-to-solvent mass ratio. We developed a support vector regression (SVR) model to predict solvent retention using these six variables, as it describes good nonlinear and high-dimensional relationships in limited datasets. Feature importance was evaluated using leave-one-out (LOO) analysis and revealed a negligible contribution of the Flory–Huggins interaction parameter, while solvent vapor pressure and polymer molecular weight dominate solvent retention. This work establishes a predictive framework for solvent retention that can be integrated into solvent screening workflows for STRAP, thereby reducing the energy required to remove the residual solvent.

## 2 Experimental Section

### 2.1 Materials

The resins used in this study were polypropylene (PP-L; F350HC2, Braskem), polypropylene (PP-M; H521, Braskem), polypropylene (PP-H; F006EC, Braskem), low-density polyethylene (LDPE; EG412, Westlake), high-density polyethylene (HDPE; Marlex HHM5502BN, Chevron Philips), poly­(ethylene terephthalate) (PET; ES306030, GoodFellow), and polystyrene (PS; 182427, Sigma-Aldrich). The solvents used were dodecane (a mixture of isomers, Thermo Scientific Chemicals) and *n*-decane (≥95%, Sigma-Aldrich), 1-decanol (≥98%, Sigma-Aldrich), dimethyl sulfoxide (DMSO; ACS reagent, 99.5%, Sigma-Aldrich), and γ-valerolactone (GVL; ReagentPlus, ≥99%, Sigma-Aldrich). The solvents also included 1,2,4-trichlorobenzene (TCB; ReagentPlus, ≥99%, Sigma-Aldrich) and aniline (ACS reagent grade, ≥99.5%, Sigma-Aldrich). Xylene was received from VWR Chemicals.

### 2.2 STRAP Experimental Procedures

Figure 2 illustrates the experimental procedure for this study. Resins (0.5–5 g) were dissolved in 50 g of solvents in a 250 mL round-bottom flask equipped with a reflux condenser connected to a cold-water supply line. The flask was partially immersed in a hot silicone oil bath. Solvents and dissolution temperatures were selected based on computational solubility predictions, their prior use in STRAP processes, and their relevant physical and chemical properties.^6,9,10,17,19^ The hot polymer solution was cooled to yield a viscous and cloudy polymer suspension, indicating polymer aggregation. After the formation of polymer aggregates, the suspension was vacuum-filtered using filter paper with 10 μm pore size for 20 min to form a polymer gel. The mass of this polymer gel (*m*_gel_) was measured. The gel was then dried in a vacuum oven at 100–130 °C for 2 h, and the mass of the final resin (*m*_resin_) was recorded. Thermogravimetric analysis (TGA) was performed on samples dried under each condition to confirm that no residual solvent remained after vacuum filtration and drying.

**Figure 2:**
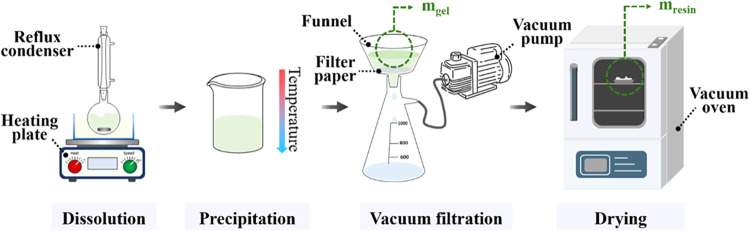
Experimental procedure for STRAP.

The solvent retention refers to the percentage mass of the solvent remaining in the polymer gel after filtration. Solvent retention was calculated according to eq 1

1 $$s\mathrm{olvent retention}\;\left(\%\right)=\frac{m_{\mathrm{gel}}-m_{\mathrm{resin}}}{m_{\mathrm{gel}}}\times 100$$

### 2.3 Cooling Rate-Controlled Precipitation Setup

We built a jacketed vessel connected to a chiller (Figure S1). Water left the chiller at a set temperature, passed through the jacket surrounding the vessel, and then returned to the chiller to maintain a constant temperature inside the vessel. After the polymer solution containing 2 g of LDPE and 150 g of xylene was transferred into the jacketed vessel, a temperature probe continuously recorded the temperature of the hot solution over time. The initial cooling rate was determined by linearly fitting the temperature profile during the first 100 s.

### 2.4 Wide-Angle X-ray Scattering (WAXS) Measurements

Precipitated polymer samples at different initial cooling rates were prepared by filtration and drying. The dried resins were placed on a powder sample holder that has 15 slots, each with 2.5 mm diameter. Kapton polyimide windows of 12.5 μm thickness were used to seal the slots. Measurements were conducted under a vacuum at ambient temperature using a Xeuss 3.0 system with an exposure time of 60 s. The scattering patterns were corrected by subtracting the background scattering from the Kapton window by using XSACT PRO advanced data analysis software. The crystallinity (%) was determined from the area ratio of crystalline peaks to total scattering area after peak fitting.^22^

### 2.5 Determination of Input Variables

Six independent variables were used as input features for the SVR model. Polymer molecular weights (*M*_w_) were determined either from manufacturer-provided datasheets or measured by gel permeation chromatography. Solvent vapor pressure values at 25 °C (*P*_vap_) were acquired from the NIST Chemistry WebBook.^23^ Hansen solubility parameters (δ_D_, δ_H_, and δ_P_) for solvents were taken from the published reference handbook.^24^ The total solvent Hansen solubility parameter ( $\delta_{T}$ ) was calculated using eq 2

2 $$\delta_{T}=\sqrt{\delta_{D}^{2}+\delta_{H}^{2}+\delta_{P}^{2}}$$

Flory–Huggins parameters (χ) were determined by eq 3, where *V* is the molar volume of the monomer repeat unit at the absolute temperature *T* and *R* is the gas constant.^25,26^ The solubility parameters of the polymers (δ_polymer_) were obtained from previously tabulated resources.^27^

3 $$\chi =\frac{V}{\mathrm{RT}}{\left(\delta_{\mathrm{polymer}}-\delta_{T}\right)}^{2}$$

The polymer-to-solvent mass ratio was determined directly from the experimental conditions, including the mass of the dissolved polymer (*m*_polymer,0_) and solvent used (*m*_solvent,0_) as shown in eq 4.

4 $$r=\frac{m_{\mathrm{polymer},0}}{m_{\mathrm{solvent},0}}$$

The predicted solubility (*S*_pred_) of PP, LDPE, HDPE, PET, PC, and PS was obtained using molecular models developed in our previous work.^4,11,28,29^ Polymer solubilities in the solvents of interest were predicted using the conductor-like screening model for realistic solvents (COSMO-RS), a combined quantum chemical and statistical mechanical approach to calculate temperature-dependent polymer solubilities in various solvent systems.^11,29–31^ The reported *S*_pred_ values are the solubility of the polymer in the solvent at the dissolution temperature. Additional details of the COSMO-RS methodology are provided in the Supporting Information.

### 2.6 Prediction of Solvent Retention

Solvent retention was predicted using machine learning (ML) regression models implemented in the scikit-learn library (v1.3.0).^32^ Data and the code are available in the Supporting Information. Three feature sets were evaluated: a six-feature model incorporating *M*_w_, *P*_vap_, δ_T_, χ, *S*_pred_, and *r*; a five-feature model excluding χ; and a four-feature model excluding χ and *r*. Feature selection for the five- and four-feature models was performed by using a leave-one-out (LOO) analysis in which each feature was removed individually, and the model was retrained using cross-validation. The resulting decrease in the predictive performance was used as a quantitative measure of importance of each feature. All numeric features were standardized using *z*-score normalization to ensure consistent feature scales during model training.

The dataset consisted of *n* = 25 unique experimental polymer–solvent combinations. Replicate experiments for a representative set of systems were performed to assess experimental reproducibility, and the error was within ±5 wt % of solvent retention. To avoid overweighting any single condition, only one representative data point from each set of replicates was used for model training and validation.

Six regression algorithms were evaluated for each feature set: support vector regression (SVR) with the radial basis function (RBF) kernel, Random Forest, Gradient Boosting Machine, Ridge Regression, ElasticNet, and K-Nearest Neighbors (KNN).^33–38^ This systematic comparison across 18 model–feature combinations (6 algorithms × 3 feature sets) enabled the identification of the best model architecture among those tested, while assessing the incremental predictive value of additional features.

Model training and validation were performed using fourfold stratified cross-validation. To mitigate sampling bias in the target variable, stratified sampling was applied using a threshold of 60% solvent retention, partitioning the dataset into low-retention (<60%) and high-retention (≥60%) groups. This stratification ensured balanced representation of both solvent retention regimes across all folds, which is critical for the small sample size. Model performance was evaluated by using the coefficient of determination (*R* ^2^), mean absolute error (MAE), and root-mean-squared error (RMSE). All metrics were computed on out-of-fold predictions to assess generalization performance.

Hyperparameters were optimized via an exhaustive grid search with cross-validated performance evaluation. For SVR models, the parameter space included the regularization constant (*C*), ε-insensitive loss (ε), and RBF kernel width (γ), tuned separately for each feature set. Optimal parameters were as follows: six-feature (*C* = 90, ε = 0.01, γ = 0.5), five-feature (*C* = 80, ε = 0.01, γ = 0.55), and four-feature (*C* = 80, ε = 0.01, γ = 0.55). Random Forests were tuned over tree count, depth, and minimum sample constraints, while Gradient Boosting additionally varied the learning rate and subsampling fraction. Ridge and ElasticNet models optimized regularization strength (α) and, for ElasticNet, the L1-ratio is characterized. KNN models varied the neighborhood size, distance weighting, and metric.

### 2.7 Correlation Analysis

Complementary univariate analysis was conducted to assess the relationship between individual features and solvent retention using Pearson and Spearman correlation coefficients. Pearson correlation was used to quantify the strength of linear relationships between solvent retention and each input, while Spearman correlation was used to assess monotonic trends independent of linearity.

### 2.8 Model Selection

Final model selection balanced predictive performance against model complexity using Akaike’s information criterion (AIC) and the Bayesian information criterion (BIC) as shown in eqs 5 and 6. In these equations, *n* is the sample size, *k* represents the model complexity (number of support vectors plus hyperparameters for SVR; effective degrees of freedom for other models), and MSE is the mean-squared error.^39^ AIC applies a constant complexity penalty (2*k*), while the BIC’s penalty (*k* ln *n*) grows with sample size, favoring simplicity more strongly. Lower AIC/BIC values indicate a superior model performance after penalization for complexity. The mean-squared error was computed from model residuals, assuming independent, normally distributed errors with zero mean and constant variance according to eq 7, where *y*_i_ and $\hat{y_{i}}$ represent the observed and predicted solvent retention values for sample *i*, respectively.

5 AIC=n⁡ln(MSE)+2k

6 BIC=n⁡ln(MSE)+k⁡ln(n)

7 $$\mathrm{MSE}=\frac{1}{n}\sum_{i=1}^{n}{\left(y_{i}-{ŷ}_{i}\right)}^{2}$$

Overall, this modeling approach provides a systematic framework for comparing feature sets and algorithms, while ensuring robust performance through stratified cross-validation and multimetric evaluation.

## 3 Results and Discussion

### 3.1 Effect of the Cooling Rate on Solvent Retention

In the STRAP process, the cooling rate and vacuum filtration time are process parameters that can influence the kinetics of phase separation and solvent removal. These kinetic factors were first examined to determine the role they play in solvent retention prior to analyzing polymer- and solvent-specific parameters.

Our first hypothesis was that the cooling rate could influence solvent retention in gels after vacuum filtration. Previous studies have shown that slower cooling enhances nucleation and growth, yielding precipitated polymers with larger and more ordered crystalline domains, which exclude solvent molecules as chains pack into tightly ordered structures.^40–44^ A higher degree of crystallinity is expected to reduce the amount of solvent retained in gels because crystalline regions incorporate minimal solvent. In addition, the cooling rate can influence the micropore size, with faster cooling resulting in a decreased pore size, potentially making the removal of the entrapped solvent more difficult.^45^

A jacketed vessel was designed to control the cooling process (Figure S1). LDPE was selected as a major component of plastic waste, and xylene has been used extensively in STRAP to dissolve LDPE. Polymer solutions were prepared by dissolving 2 g of LDPE dissolved in 150 g of xylene at 100 °C. The resulting hot solutions were precipitated in the jacketed vessel. Figure 3a shows the temperature profiles of the hot polymer solution cooled isothermally at 35, 60, and 80 °C, and the initial 100 s were used to determine initial cooling rates. The rates were calculated as 0.047, 0.080, and 1.171 °C s^–1^, respectively, which are comparable to those used in previous polymer crystallization (0.1–1 °C s^–1^).^43,44^ Lower bath temperature resulted in faster cooling, and the solution temperature reached a steady state with the vessel after 2000 s.

**Figure 3:**
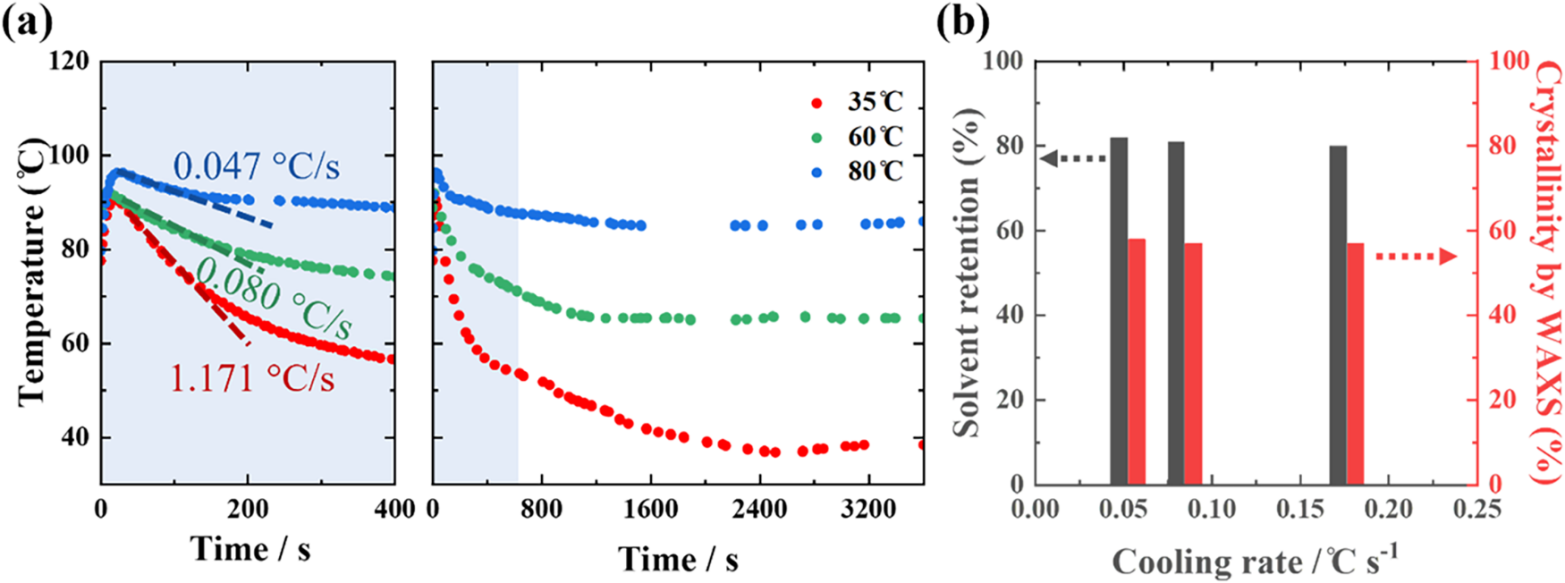
(a) Temperature profiles of hot polymer solution cooled isothermally at different vessel temperatures. The shaded region was used to determine the initial cooling rate. (b) Solvent retention and crystallinity as functions of the initial cooling rate.

After solvent removal, solvent retention and crystallinity were measured as functions of the initial cooling rate (Figure 3b). Both remained nearly constant, with solvent retention at approximately 80% and crystallinity at approximately 55%. It is important to note that crystallinity was measured after all samples were dried under identical conditions in the oven, resulting in the same thermal histories. No difference in crystallinity was observed from different precipitations likely because the polymers had a similar thermal history. The similar solvent retention observed at different initial cooling rates for the LDPE–xylene system suggests that the initial cooling rate has only a minor influence on solvent retention in gels.

Precipitation by the addition of an antisolvent was examined as a kinetic extreme case to determine whether rapid phase separation affects solvent retention in gels.^7^ Hot polymer solutions containing LDPE and xylene were cooled by adding 100 g of either acetone, methanol, or isopropyl alcohol, water as an antisolvent. Solvent retention values of 78–80% were measured for samples precipitated using the antisolvents and water (Table S1), which are comparable to the ∼80% obtained from cooling rate-controlled precipitation. These results demonstrate that the precipitation method has little impact on solvent retention in gels for the LDPE-xylene system in dissolution-based plastic recycling. The reverse antisolvent experiment was also performed by slowly adding the LDPE–xylene solution into acetone. A white suspension was observed, while vacuum filtration using a 10 μm filter paper did not recover any solid polymer. This suggests that the precipitated polymer particles were smaller than the filter pore size and therefore passed through the filter, which is consistent with the literature showing that depending on the mixing conditions, the polymer particle size can vary.^16^ Previous studies have reported minimal changes in molecular weight distribution before and after polymer recycling with STRAP technology.^46^

### 3.2 Effect of Vacuum Filtration Time on Solvent Retention

The effect of the vacuum filtration time on solvent retention was evaluated to determine whether extending filtration promotes further removal of free or loosely bound solvent prior to drying. Decanol was selected as the solvent for this experiment because of its low vapor pressure, which minimizes solvent loss by evaporation during prolonged vacuum filtration. This allowed us to decouple the effect of vacuum filtration on solvent retention from that of evaporation due to a high vapor pressure. In addition, PP-decanol resulted in the highest solvent retention, facilitating the measurement of changes in solvent retention as a function of the filtration time.

As shown in Table 1, solvent retention was approximately 84% after 20 min of vacuum filtration and remained essentially unchanged after 360 min for PP in decanol at a polymer-to-solvent ratio of 0.05. The negligible change in solvent retention with an extended filtration time indicates that most free solvent was removed within the first 20 min with the remaining solvent trapped within the polymer gel. Therefore, 20 min of vacuum filtration was considered sufficient and was used in all subsequent experiments. For solvents with higher vapor pressures, an extended filtration time might lead to additional solvent removal through evaporation. In such systems, a slight decrease in solvent retention at long filtration times could arise from the volatility of the solvent rather than from vacuum filtration.

**Table 1:** Solvent Retention of PP in Decanol with Different Vacuum Filtration Times^a^

polymers	*M*_w_ (g/mol)	solvents	*r*	filtration time (min)	solvent retention(%)
PP	250,000	decanol	0.05	5	89.24
PP	250,000	decanol	0.05	10	87.19
PP	250,000	decanol	0.05	20	83.75
PP	250,000	decanol	0.05	360	85.22

a Solvent retention was measured at an initial polymer-to-solvent ratio (*r*) of 0.05 after 20 and 360 min of filtration.

### 3.3 Solvent Retention in Different Polymer Gels

In Sections 3.1 and 3.2, process-related kinetic factors, including cooling rate and vacuum filtration time, were shown to have little influence on solvent retention under the conditions investigated. Attention was then shifted to polymer- and solvent-specific parameters to examine their effect on the solvent retention in gels for a broad range of polymer–solvent combinations.

Table 2 presents experimental data from a total of 34 experiments, corresponding to 25 unique conditions for measuring solvent retention using six polymers and nine solvents. Out of the 25 unique experimental conditions, seven were randomly selected and measured in replicate, resulting in standard deviations within 3%, indicating good experimental reproducibility. The polymers used in this work are PP, LDPE, HDPE, PET, PC, and PS, while the solvents are xylene, decalin, aniline, TCB, dodecane, decane, decanol, GVL, and DMSO. HDPE and LDPE were selected to investigate the effect of branching on the solvent retention. PP was included to study the influence of the side chain symmetry and semicrystalline structure. PET was chosen as a widely used semicrystalline commodity polyester, while PC and PS were selected as amorphous polymers. These polymer–solvent pairs were selected based on the ability of each solvent to selectively dissolve each polymer, with solvents having a range of different boiling points and predicted solubilities. A range of different polymer-to-solvent ratios were tested as the polymer concentration has been shown to impact viscosity during the filtration.^47^ The distribution of input feature values is presented in the box plot in Figure S2. This confirms that the experimental conditions cover a broad range of input feature values in dissolution-based recycling.

**Table 2:** Solvent Retention in Gels and Six Variables^a^

experiment	polymer	solvent	*r*	*M*_w_ (g mol^–1^)	*P*_vap_(Pa, 25 °C)	δ_T_ (MPa^0.5^)	*S*_pred_ (wt %)	χ	solvent retention (%)
1	PP-L	xylene	0.01	250,000	879	17.9	25.93	0.0152	20.16
2a	PP-L	xylene	0.05	250,000	879	17.9	25.93	0.0152	36.69
2b	PP-L	xylene	0.05	250,000	879	17.9	25.93	0.0152	40.37
2c	PP-L	xylene	0.05	250,000	879	17.9	25.93	0.0152	39.44
3	PP-M	xylene	0.05	423,000	879	17.9	2.40	0.0152	59.85
4	PP-H	xylene	0.05	821,000	879	17.9	10.89	0.0152	52.30
5	PP-L	xylene	0.1	250,000	879	17.9	25.93	0.0152	47.98
6a	PP-L	dodecane	0.05	250,000	18	16	23.40	0.0189	48.61
6b	PP-L	dodecane	0.05	250,000	18	16	23.40	0.0189	52.53
7	PP-M	dodecane	0.05	423,000	18	16	8.26	0.0189	62.52
8	PP-H	dodecane	0.05	821,000	18	16	18.84	0.0189	79.51
9a	PP-L	decane	0.05	250,000	190	15.7	27.67	0.0319	49.21
9b	PP-L	decane	0.05	250,000	190	15.7	27.67	0.0319	55.34
10a	PP-L	decanol	0.05	250,000	1.13	19.4	11.89	0.1127	83.75
10b	PP-L	decanol	0.05	250,000	1.13	19.4	11.89	0.1127	79.47
11	PP-L	decalin	0.05	250,000	104.8	18	8.84	0.0189	82.31
12	PP-L	TCB	0.05	250,000	61.33	20.9	23.97	0.2868	85.60
13a	LDPE	xylene	0.05	86,500	879	17.9	39.51	0.0354	35.56
13b	LDPE	xylene	0.05	86,500	879	17.9	39.51	0.0354	36.94
13c	LDPE	xylene	0.05	86,500	879	17.9	39.51	0.0354	39.45
14	LDPE	dodecane	0.05	86,500	18	16	57.46	0.0005	73.07
15a	LDPE	decane	0.05	86,500	190	15.7	61.67	0.0031	78.27
15b	LDPE	decane	0.05	86,500	190	15.7	61.67	0.0031	75.44
16	LDPE	decanol	0.05	86,500	1.13	19.4	46.88	0.1291	67.18
17	HDPE	xylene	0.05	143,800	879	17.9	18.97	0.0232	30.32
18	HDPE	dodecane	0.05	143,800	18	16	39.79	0.003	85.79
19a	HDPE	decane	0.05	143,800	190	15.7	44.00	0.0076	82.43
19b	HDPE	decane	0.05	143,800	190	15.7	44.00	0.0076	85.45
20	HDPE	decanol	0.05	143,800	1.13	19.4	29.25	0.1027	86.35
21	PET	DMSO	0.05	35,000	79.99	26.7	30.41	2.1421	92.33
22	PET	aniline	0.05	35,000	88.92	22.5	25.06	0.2255	89.38
23	PET	GVL	0.05	35,000	31.33	23.2	40.11	0.4126	91.65
24	PC	DMSO	0.05	49,218	79.99	26.7	17.24	3.4742	84.30
25	PS	DMSO	0.05	280,000	79.99	26.7	22.47	2.3182	73.98

a A total of 34 experiments were conducted, corresponding to 25 unique conditions. Experiments labeled with the same number indicated replicate measurements. The standard deviations of solvent retention were within 3%, indicating good experimental reproducibility.

Clear differences in solvent retention were observed between amorphous and semicrystalline polymers. Amorphous polymers such as PC and PS consistently exhibited high solvent retention exceeding 74% (experiments 21–25), while semicrystalline polyolefins such as PP, LDPE, and HDPE generally showed lower retention under comparable conditions. In contrast, the effect of polymer branching is not apparent in the system investigated. Although LDPE exhibits a slightly higher retention than that of HDPE in xylene (experiments 13 and 17), this trend is reversed in low-volatility solvents such as decane and dodecane, where HDPE shows higher retention (experiments 15 and 19 and experiments 14 and 18).

Figure 4a shows a box-and-scatter plot of the dataset in Table 2 for the 25 unique experimental conditions. Solvent retention spans 20.16–91.65% with the first quartile (Q1) at 49.21% and the third quartile (Q3) at 84.3%. The median retention (73.98%) is greater than the mean (67.16%), indicating that the dataset is slightly weighted toward higher solvent retention values. Retention values below Q1 were observed primarily when xylene was used as the solvent (Table 2). Because xylene contains an aromatic ring, this initially raised the question of whether the molecular structure contributed to its lower retention. To test this, other ring-containing solvents, decalin and TCB, were also evaluated. Both exhibited relatively high retention (82.31 and 85.60%, respectively), indicating that xylene’s lower retention is not due to its chemical structure. Instead, the most likely cause is its much higher vapor pressure (∼879 Pa) compared with decalin (105 Pa) and TCB (61 Pa).^23^

**Figure 4:**
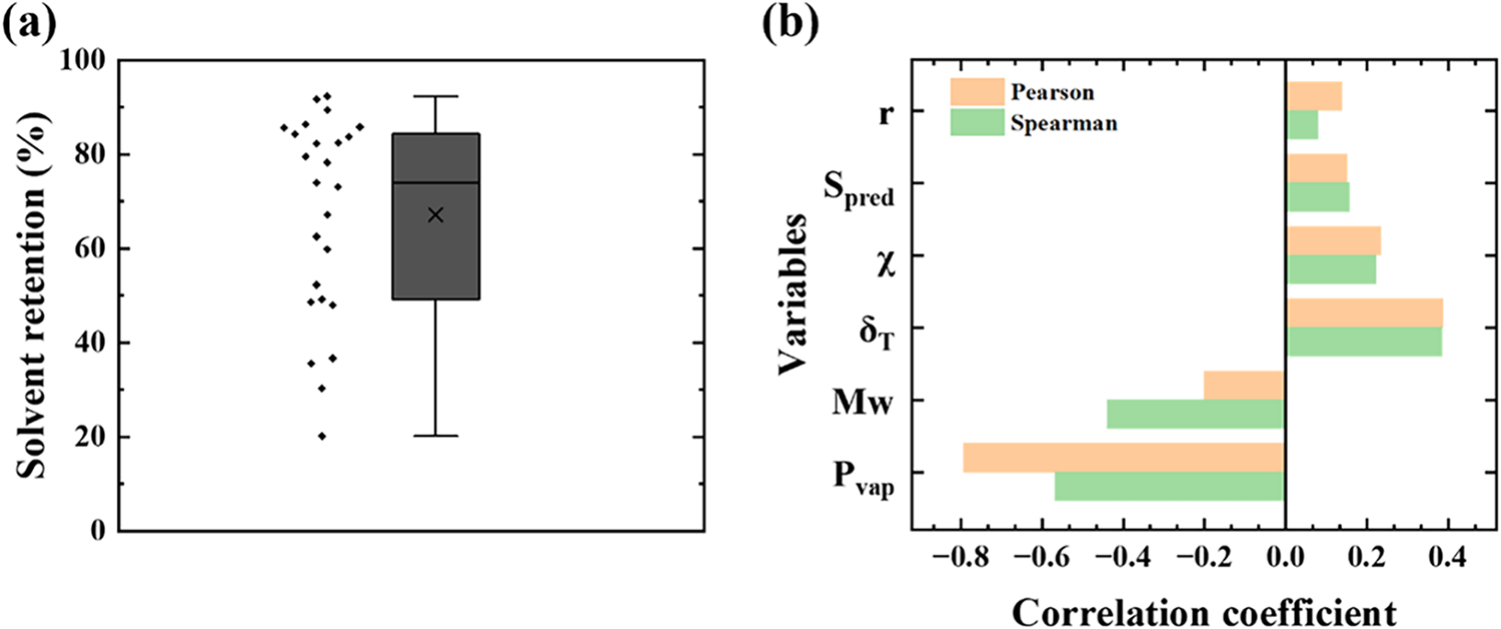
Summary of solvent retention experiments and independent variables. (a) Box-and-scatter plot of measured solvent retention. The box shows the interquartile range with the median line; the “*x*” marks the mean; whiskers span the minimum and maximum values. The scatter shows the distribution of data. (b) Pearson and Spearman rank correlation coefficients between each variable and solvent retention.

Clear trends are not apparent from Table 2. Therefore, six variables were correlated to solvent retention using Pearson and Spearman correlation analyses, as shown in Figure 4(b), to determine if any single parameter exhibits a strong monotonic or linear relationship. The six variables are *P*_vap_, δ_T_, χ, *S*_pred_, *r*, and *M*_w_. *P*_vap_ is the solvent vapor pressure and describes solvent volatility, and δ_T_ is the total Hansen solubility parameter represented by the combined contribution of dispersion, polar, and hydrogen bonding interactions. The polymer–solvent affinity is described by the Flory–Huggins interaction parameter χ, the predicted solubility using COSMO-RS is represented by *S*_pred_, the polymer–solvent ratio *r* defines the initial concentration and viscosity of the polymer solution, and the *M*_w_ determines the chain length and segmental mobility. *M*_w_ may influence solvent retention through chain entanglements, which restricts solvent diffusion in a gel.^48^

In Figure 4b, *P*_vap_ exhibits a strong negative Pearson correlation (−0.8), indicating that *P*_vap_ is the single most strongly associated variable with solvent retention in an approximately linear manner, with higher vapor pressure solvents tending to give lower retained solvent. Figure S3 further shows that *P*_vap_ exhibits the strongest linearity among the six features. The Spearman correlation for *P*_vap_ is slightly weaker (−0.55), suggesting that although the overall trend is monotonic, the relationship is not perfectly linear across all data points. *M*_w_ also shows a moderate negative correlation, with Spearman (−0.40) being stronger in magnitude than Pearson (−0.25). This discrepancy implies a monotonic but nonlinear relationship between polymer molecular weight and solvent retention with larger *M*_w_ polymers generally retaining less solvent.

For δ_T_, χ, *S*_pred_, and *r*, both the Pearson and Spearman coefficients remain comparatively small. In several cases, Spearman exceeds Pearson, suggesting that these variables may have weak but monotonic associations that are not well captured by linear correlation alone. Overall, the differences between Pearson and Spearman correlations in Figure 4b indicate that *P*_vap_ has both the strongest and most linear relationship with solvent retention, whereas other variables show weaker, possibly nonlinear monotonic trends, reinforcing the conclusion that solvent retention arises from a nonlinear and multivariate interplay rather than a single controlling factor.

A single case study using PP was conducted to examine several factors (Figure S3). The relationship between the *M*_w_ and solvent retention was examined using PP-L, PP-M, and PP-H in dodecane at a fixed polymer-to-solvent ratio of 0.05. As shown in Figure S3­(a), solvent retention increases approximately linearly with *M*_w_. For *P*_vap_, a monotonic relationship is observed in which solvent retention decreases as vapor pressure increases (Figure S3­(b)). Lastly, *r* also shows a linear relationship with solvent retention (Figure S3­(c)). These single polymer subsets, therefore, suggest that the observed relationships are not simply artifacts of combining different polymer types.

However, when the broader dataset including multiple polymer systems is considered, no single factor alone fully explains solvent retention for different polymer–solvent systems, as shown in Figure 5. Instead, solvent retention is influenced by the combined effect of multiple factors. This complexity motivated the use of ML to identify the governing factors for solvent retention and develop a predictive model.

**Figure 5:**
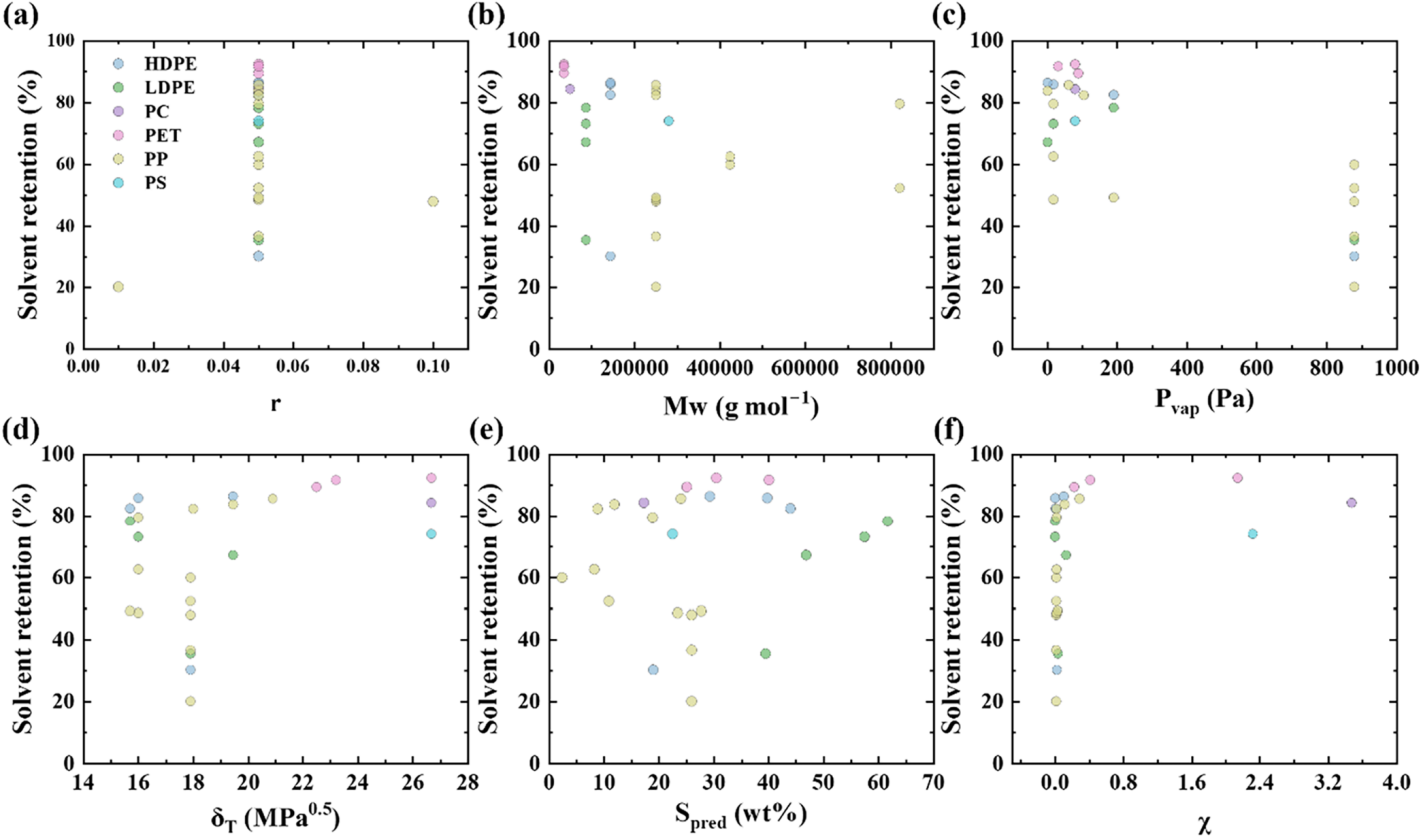
Solvent retention as a function of (a) *r*, (b) *M*_w_, (c) *P*_vap_, (d) δ_T_, (e) *S*_pred_, and (f) χ for different plastic types.

We evaluated six supervised learning algorithms to identify an appropriate regression model for solvent retention, SVR (with a nonlinear RBF kernel), Ridge, ElasticNet, Random Forest, Gradient Boosting, and KNN across three feature sets, Figure S4. Test *R* ^2^ values ranged from 0.56 to 0.78. The nonlinear methods (KNN and SVR) consistently achieved the highest performance, with SVR reaching a test *R* ^2^ of 0.78 for the 5-feature set and KNN yielding *R* ^2^ of 0.74, indicating that models designed for nonlinear relationships perform best. Tree-based methods (Random Forest and Gradient Boosting) showed intermediate performance, while linear models (Ridge and ElasticNet) performed worse overall. We used SVR, which is well suited for relatively small datasets with nonlinear behavior, for further analysis. SVR provides a partial dependence of solvent retention on a single variable (Figure S5). All models were trained and assessed using stratified cross-validation, with solvent retention binned at a 60% threshold to maintain balanced representation of low- and high-retention samples in each split.

Feature selection was evaluated using a LOO approach in which each variable was removed individually, and the cross-validated *R* ^2^ was recalculated, as shown in Figure 6. For the six-feature SVR, an *R* ^2^ of 0.70, RMSE of 9.4, and MAE of 11.0 were obtained (Figure 6a,6b). For the five-feature model, removing χ produced the highest performance (*R* ^2^ = 0.78; RMSE = 8.4; MAE = 9.7), indicating that χ adds noise rather than a useful signal and that the model improves when it is excluded (Figure 6c,d).

**Figure 6:**
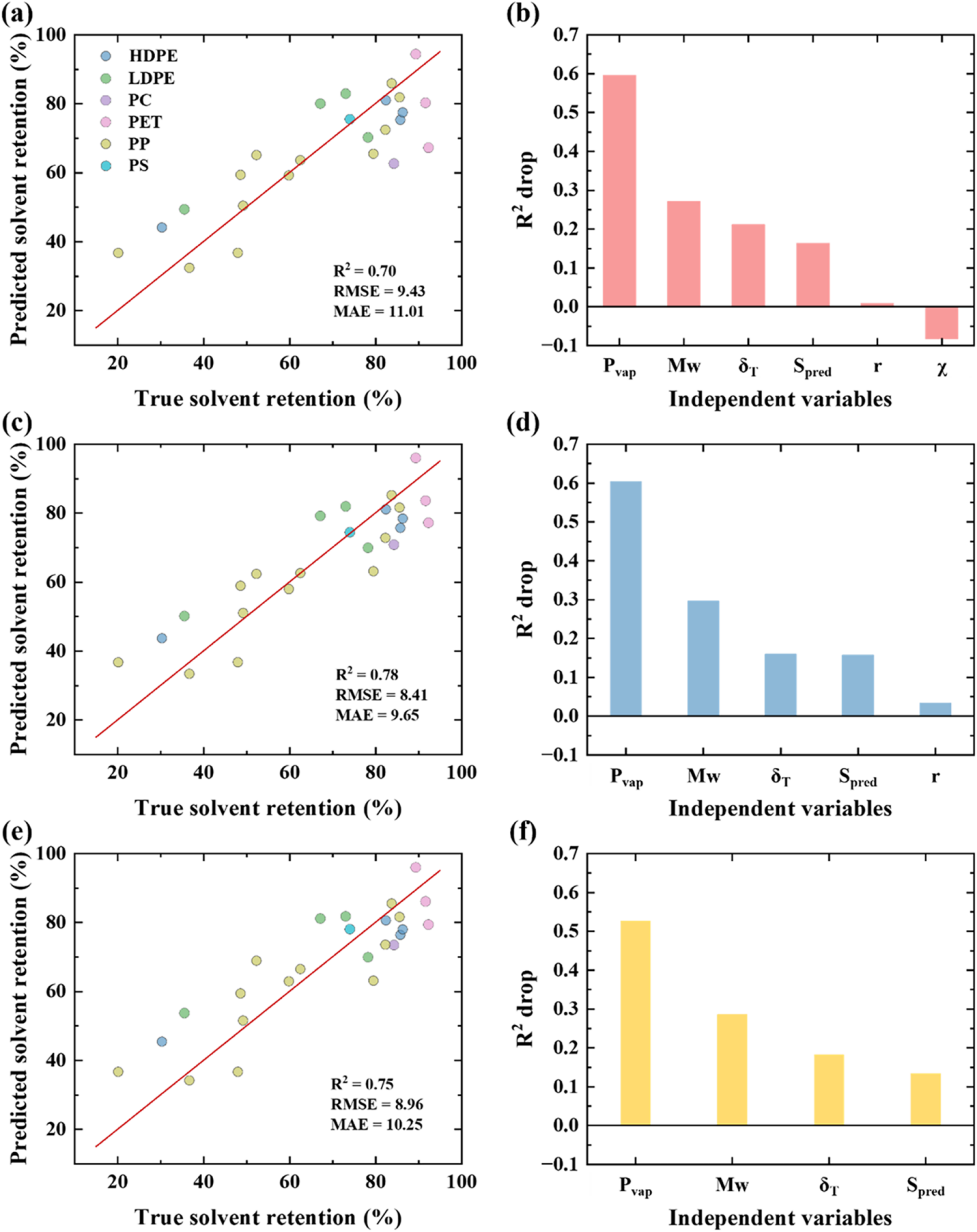
Model performance and feature contribution with different numbers of independent variables using SVR. The parity plots (a, c, e) and LOO *R* ^2^ drop analyses (b, d, f) correspond to models trained with six, five, and four features, respectively.

The improvement upon removal of χ can be explained both statistically and physically. χ is highly correlated with δ_T_ according to eq 3. The redundancy in nonlinear models can worsen generalization and decrease model predictive performance. Second, the χ values used in this work may not fully reflect the polymer–solvent interaction, which can limit its predictive contribution.^49,50^ The χ values were estimated using Hildebrand solution theory, which is a heuristic model based on cohesive energy density and does not explicitly account for the specific nature of polymer–solvent interactions.^51^ When the five-feature model was trained using χ instead of δ_T_, the model performance deteriorated substantially (*R* ^2^ = 0.49), further indicating that χ estimated using Hildebrand solution theory has a limited ability to represent the polymer–solvent interactions that impact solvent retention in dissolution-based recycling processes.

In contrast, removing *P*_vap_ caused the largest decrease in *R* ^2^, confirming that the solvent vapor pressure is the most influential predictor, followed by *M*_w_ and δ_T_. A second round of LOO analysis on the four-feature model showed that performance was maximized when r was removed (*R* ^2^ = 0.75; RMSE = 10.3; MAE = 8.9), while removing *P*_vap_ again resulted in the largest performance drop (Figure 6e,6f). Together, these results demonstrate that χ and r contribute little to predictive accuracy, whereas *P*_vap_ remains the dominant variable, with *M*_w_ acting as a meaningful secondary contributor.

Table 3 summarizes the AIC and BIC used to assess the trade-off between model fit and complexity for the six-, five-, and four-feature SVR models. Both criteria decrease moderately for the five-feature model relative to the six-feature model (ΔAIC = −7.26; ΔBIC = −7.27), indicating that removing χ improves the overall model quality by reducing unnecessary complexity, while enhancing predictive performance. The four-feature model shows a slight increase in AIC and BIC (ΔAIC = 2.59; ΔBIC = 2.60) compared with the five-feature model, further supporting our choice of the five-feature model.

**Table 3:** Models with Five and Six Features and Their Fitness^a^

features	DOF*	R^2^	AIC	BIC
*P*_vap_, δ_T_, *M*_w_, *S*_pred_, *r*, and χ	6	0.70	179.09	214.44
*P*_vap_, δ_T_, *M*_w_, *S*_pred_, and *r*	5	0.78	171.83	207.17
*P*_vap_, δ_T_, *M*_w_, and *S*_pred_	4	0.75	174.42	209.77

a DOF*: degree of freedom.

The five-feature SVR model is identified as the best model of those tested, permitting analysis of the physical contribution of each feature based on Figure 6(d). *P*_vap_ has the largest impact and therefore plays the primary role in controlling solvent retention by influencing evaporation during precipitation, filtration, and drying. While solvents with high *P*_vap_ are more easily removed and tend to result in lower solvent retention in gels, these solvents often have lower flash points, which introduce additional safety considerations for large-scale operation. This result suggests that the final retained solvent content is controlled not only by how much solvent is initially trapped during phase separation but also by how easily the retained solvent can subsequently escape from the swollen polymer matrix. *M*_w_ is the second most important feature and governs transport kinetics within the polymer matrix. The diffusion coefficient of the solvent through a polymer matrix is inversely proportional to *M*_w_, indicating that higher *M*_w_ slows down solvent diffusion and hinders mechanical solvent removal.^52^ The contributions of δ_T_ and *S*_pred_ are comparable. δ_T_ represents the cohesive energy density of the solvent, which describes the strength of solvent–solvent interactions.^24^ *S*_pred_ reflects the thermodynamic affinity between the polymer and the solvent. In contrast, *r* shows the smallest effect on solvent retention. This limited contribution is attributed to the narrow distribution of r values, where most experiments were conducted at *r* = 0.05 (Figure S2a). Together, the dominant contributions of *P*_vap_ and *M*_w_ suggest that solvent retention is governed primarily by solvent volatility and transport limitations within the polymer matrix rather than by thermodynamic affinity alone.

## 4 Conclusions

This study provides a fundamental understanding of solvent retention in polymer gels formed during STRAP. We identified the key factors that play dominant roles in determining solvent retention by combining experimental measurements with data-driven ML modeling.

Process-related kinetic factors, including cooling rate, vacuum filtration time, and the addition of an antisolvent, were found to have minimal influence on solvent retention. The LDPE-xylene gels were shown to have 80% solvent retention at different initial cooling rates. Similarly, extended filtration led to negligible changes in solvent retention, indicating that only free solvent is removed during filtration, while the remaining solvent remains entrapped within a gel.

In this study, we measured solvent retention in 34 polymer gels comprising 8 polymers and 9 solvents, with observed retention values ranging from 20.16 to 91.65 wt % (median: 73.98 wt %). Using these data, we developed a five-feature SVR model to predict solvent retention. Solvent vapor pressure emerged as the dominant predictor, followed by *M*_w_, δ_T_, *S*_pred_, and *r*. The final model achieved an *R* ^2^ of 0.78, demonstrating strong predictive capability and providing insights into the factors determining solvent retention in gels.

Overall, this study establishes a quantitative framework for describing and predicting solvent retention in STRAP. The results show that solvent volatility plays a far more dominant role than precipitation or filtration conditions in determining residual solvent levels in gels. When combined with solvent-screening workflows, this predictive model can be used to down-select candidate solvents that not only dissolve target polymers but also exhibit low predicted solvent retention.^53^ This directly reduces the energy required for solvent removal and improves the feasibility of STRAP. In addition, in situ techniques such as dynamic light scattering during precipitation could provide mechanistic insights into gel formation in future studies.^54^

## Supplementary Material







## Notes

Predicted solubility calculations, schematic illustration of the experimental setup, model performance for six regression algorithms, and hyperparameter optimization of the SVR model (PDF)
Dataset (CSV)
Full regression code (PDF)
