# Supplementary material for: Factors Affecting Solvent Retention due to Gel Formation during Dissolution-Based Plastic Recycling
Source: ACS Sustain Chem Eng. 2026 May 2;14(19):9093–103. doi: 10.1021/acssuschemeng.5c13798 (PMC13188057; doi:10.1021/acssuschemeng.5c13798)
Supplement: Supplementary file 1 [file sc5c13798_si_001.pdf]

Supplementary Information

Factors Affecting Solvent Retention due to Gel Formation during Dissolution-Based Plastic Recycling

Hedam Kim†, Ali Altamimi‡, Whitney S. Loo†, Reid C. Van Lehn†, ‡, and George W. Huber†*

†Department of Chemical and Biological Engineering, University of Wisconsin-Madison, Madison, WI 53706, USA

‡Department of Chemistry, University of Wisconsin-Madison, WI 53706, USA

*Corresponding authors. E-mail: gwhuber@wisc.edu

Keywords

Solvent retention; solvent removal; dissolution-based recycling; STRAP™; plastic recycling; machine learning; support vector regression

This file contains:

- 11 pages
- 8 figures
- 1 table

Section S1 Predicted solubility calculations

Polymer solubilities in the solvents of interest were predicted using COSMO-RS combining quantum chemical and statistical mechanical approach to calculate temperature-dependent polymer solubilities in various solvent systems.[1,2] COSMO-RS represents each molecule based on the screening charge density, obtained from density functional theory calculations in our previous work, that arises at molecular surfaces due to polarization of the surrounding medium.[1,3–5] We approximated the screening charge densities of polymer molecules by modeling oligomer structures with deactivated terminal groups. COSMO-RS solubility calculations were performed using COSMOtherm 19 with the BP_TZVP_19 parameterization. LDPE calculations used our PE molecular model consisting of 31 conformers of a 6-mer structure with a reference melting temperature of 141.45 °C and reference solubility of 23.1 wt% at 110 °C in toluene. HDPE calculations used the same PE model with a reference melting temperature of 140 °C and reference solubility of 16.2 wt% in dodecane at 120 °C.[6] PP calculations relied on our PP model using 25 conformers of an isotactic 6-mer structure and reference melting temperature of 187.55 °C and reference solubility of 7.5 wt% in toluene obtained from a 250,000 g/mol PP resin, which was used for all PP samples considered in this work. PET calculations relied on our PET model using 22 conformers of a 4-mer structure and reference melting temperature of 279.85 °C and reference solubility of 13.3 wt% in DMSO. PS calculations relied on our PS model using 29 conformers of a 6-mer atactic structure using a reference melting temperature of 243.05 °C and a reference solubility of 41.2 wt% in toluene at 110 °C. PC calculations relied on our PC model using 19 conformers of a 4-mer structure with a reference melting temperature of 300 °C and reference solubility of 16.7 wt% in THF at 65 °C.

Section S2 Hyperparameter Optimization and Residual Diagnostics of Five-Feature SVR Model

A hyperparameter screen was performed for the SVR models and the optimal parameters for five-feature SVR model—C = 80, ε = 0.01, and γ = 0.55—were selected based on the highest cross-validated R^2 (Figure S6–8).

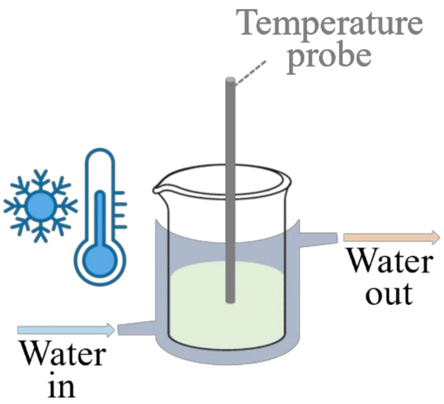

Figure S1. Schematic illustration of the jacketed vessel with two necks connected to a chiller.

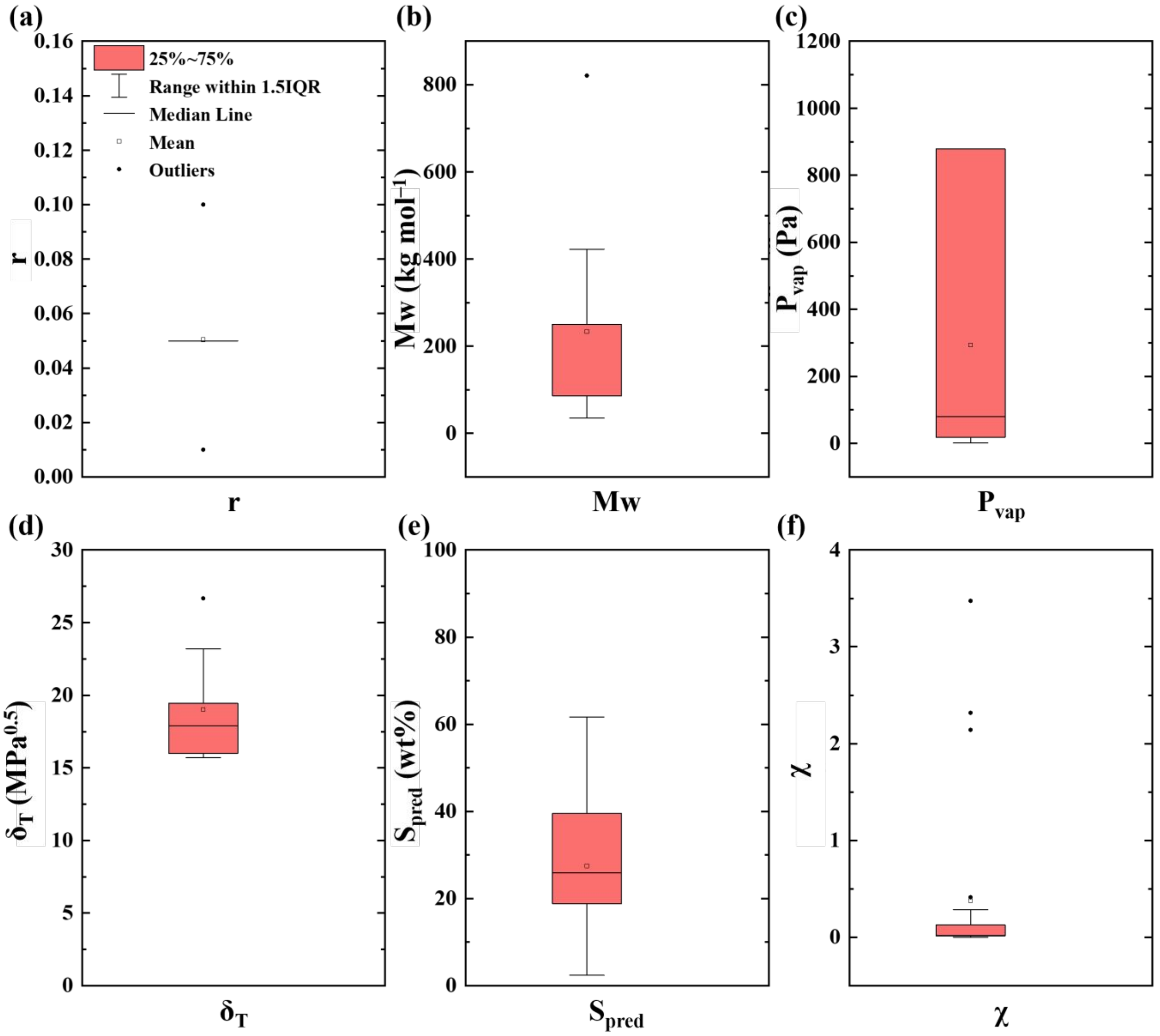

Figure S2. Box plot of input features: (a) r, (b) Mw, (c) P_{vap}, (d) δ_T, (e) S_{pred}, and (f) χ. The box shows the interquartile (IQ) range with the median line and the "▫" marks the mean. Whiskers span the minimum and maximum values. The scatter shows the distribution of data.

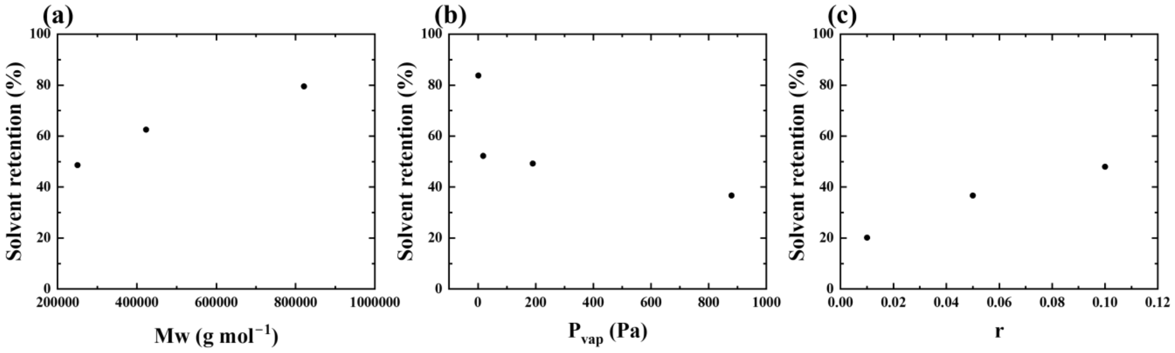

Figure S3. Solvent retention as function of (a) Mw, (b) P_{vap}, and (c) r for PP. (a) Dependence of solvent retention on Mw for PP dissolved in dodecane at r=0.05. (b) Dependence of solvent retention on P_{vap} for PP dissolved in different solvents (xylene, dodecane, decane, and decanol). (c) Effect of polymer-to-solvent ratio for PP dissolved in xylene.

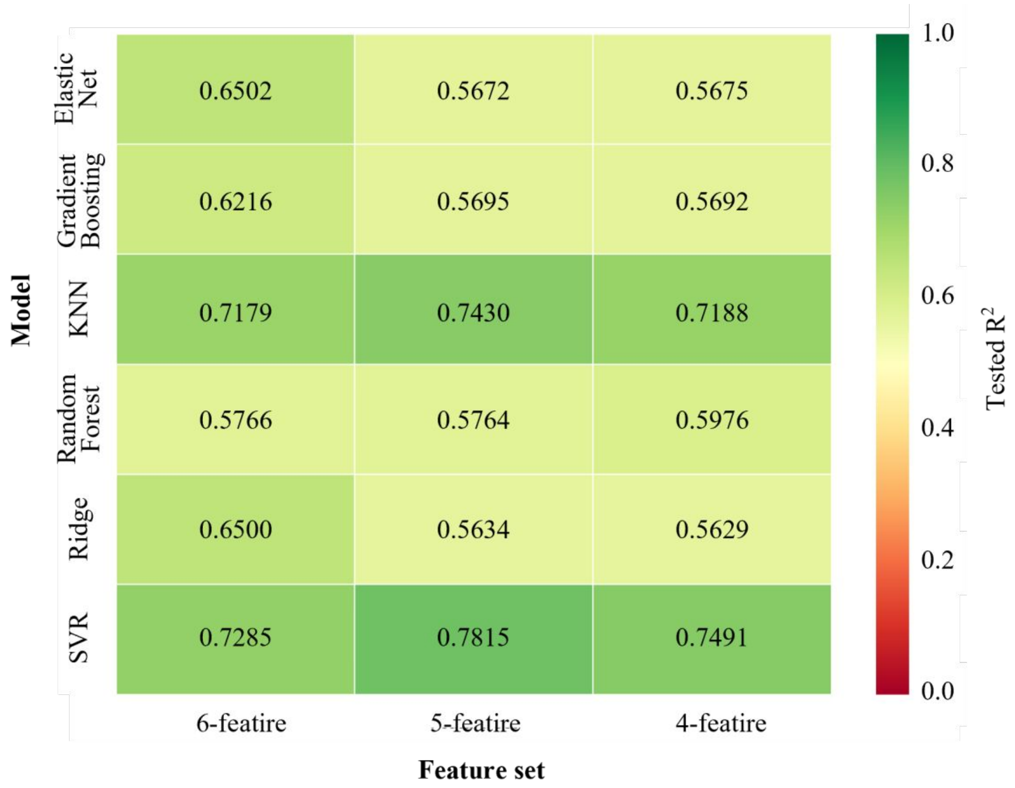

Figure S4. Model performance (Tested R^2) for six regression models evaluated using three different feature sets. SVR and KNN exhibit the highest predictive accuracy.

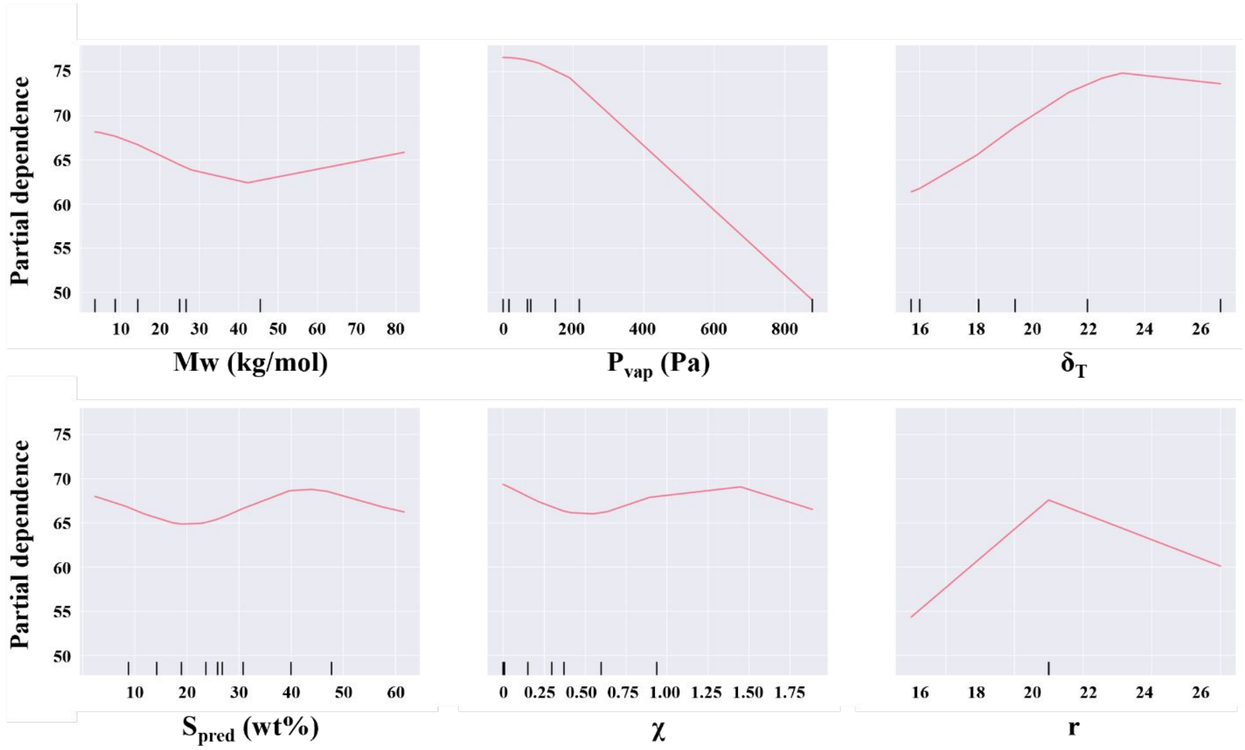

Figure S5. Partial dependence of each variable on solvent retention. P_{vap} shows the linear decrease in partial dependence in agreement with Pearson correlation.

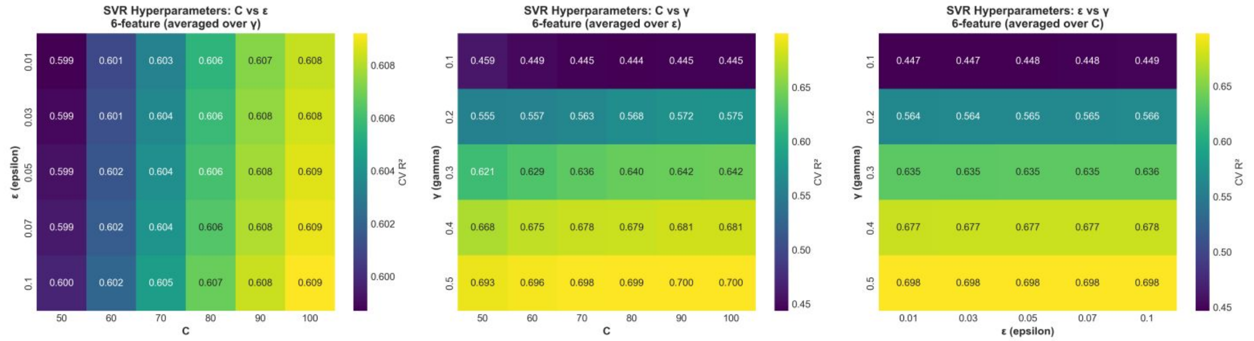

Figure S6. Hyperparameter sweeping and tested R^2 for 6-feature SVR model.

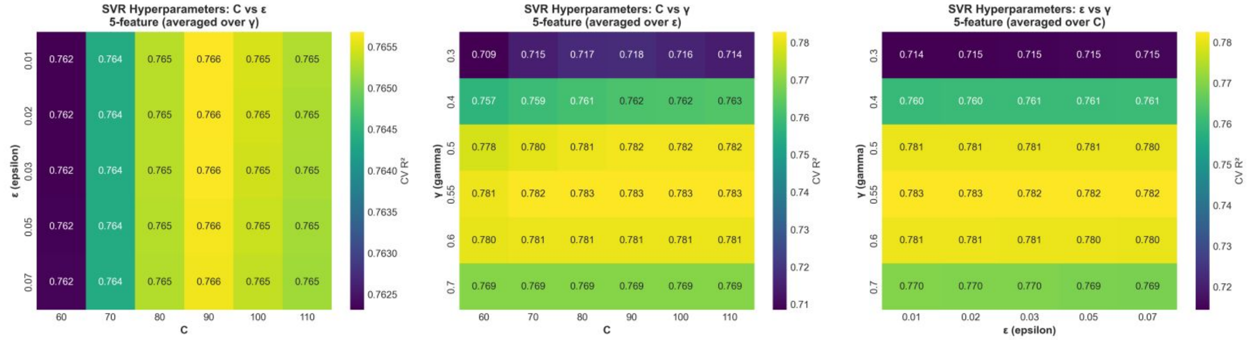

Figure S7. Hyperparameter sweeping and tested R^2 for 5-feature SVR model.

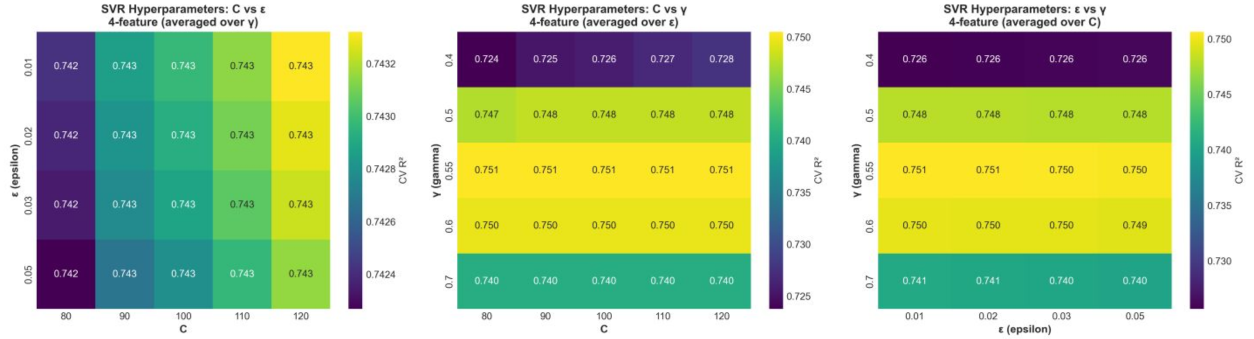

Figure S8. Hyperparameter sweeping and tested R^2 for 4-feature SVR model.

Table S1. Solvent retention when 2 g of LDPE dissolved in 150 g of xylene was precipitated by adding anti-solvents.

Anti-solvent	Solvent retention (%)
Acetone	78
Water	79
Methanol	79
Isopropyl alcohol	80

References

(1) Sánchez-Rivera, K. L.; Munguía-López, A. del C.; Zhou, P.; Cecon, V. S.; Yu, J.; Nelson, K.; Miller, D.; Grey, S.; Xu, Z.; Bar-Ziv, E.; Vorst, K. L.; Curtzwiler, G. W.; Van Lehn, R. C.; Zavala, V. M.; Huber, G. W. Recycling of a Post-Industrial Printed Multilayer Plastic Film Containing Polyurethane Inks by Solvent-Targeted Recovery and Precipitation. *Resour. Conserv. Recycl.* **2023**, *197*, 107086. https://doi.org/10.1016/j.resconrec.2023.107086.

(2) Zhou, P.; Sánchez-Rivera, K. L.; Huber, G. W.; Van Lehn, R. C. Computational Approach for Rapidly Predicting Temperature-Dependent Polymer Solubilities Using Molecular-Scale Models. *ChemSusChem* **2021**, *14* (19), 4307–4316. https://doi.org/10.1002/cssc.202101137.

(3) Klamt, A. Conductor-like Screening Model for Real Solvents: A New Approach to the Quantitative Calculation of Solvation Phenomena. *J. Phys. Chem.* **1995**, *99* (7), 2224–2235. https://doi.org/10.1021/j100007a062.

(4) Klamt, A.; Jonas, V.; Bürger, T.; Lohrenz, J. C. W. Refinement and Parametrization of COSMO-RS. *J. Phys. Chem. A* **1998**, *102* (26), 5074–5085. https://doi.org/10.1021/jp980017s.

(5) Zhou, P.; Yu, J.; L. Sánchez-Rivera, K.; W. Huber, G.; Lehn, R. C. V. Large-Scale Computational Polymer Solubility Predictions and Applications to Dissolution-Based Plastic Recycling. *Green Chem.* **2023**, *25* (11), 4402–4414. https://doi.org/10.1039/D3GC00404J.

(6) Sánchez-Rivera, K. L.; Zhou, P.; Radkevich, E.; Sharma, A.; Bar-Ziv, E.; Van Lehn, R. C.; Huber, G. W. A Solvent-Targeted Recovery and Precipitation Scheme for the Recycling of Up to Ten Polymers from Post-Industrial Mixed Plastic Waste. Social Science Research Network: Rochester, NY October 14, 2024. https://doi.org/10.2139/ssrn.4986914.

(7) Michelucci, U. Feature Importance and Selection. In *Fundamental Mathematical Concepts for Machine Learning in Science*; Michelucci, U., Ed.; Springer International Publishing: Cham, 2024; pp 229–242. https://doi.org/10.1007/978-3-031-56431-4_10.
