# Supplementary material for: Factors Affecting Solvent Retention due to Gel Formation during Dissolution-Based Plastic Recycling
Source: ACS Sustain Chem Eng. 2026 May 2;14(19):9093–103. doi: 10.1021/acssuschemeng.5c13798 (PMC13188057; doi:10.1021/acssuschemeng.5c13798)
Supplement: Supplementary file 3 [file sc5c13798_si_003.pdf]

```python
"""
Solvent Retention Machine Learning Analysis
- R² comparisons (6, 5, 4 features for all models)
- Detailed correlation analysis (Pearson & Spearman)
- Individual variable linear analysis
- Complete residual analysis for all models
- Parity plots for best models in each feature set
- AIC/BIC analysis for SVR models
- Additional visualizations
"""

import warnings
from pathlib import Path
import numpy as np
import pandas as pd
import matplotlib.pyplot as plt
import seaborn as sns
from scipy import stats
from itertools import combinations

from sklearn.preprocessing import StandardScaler, OneHotEncoder
from sklearn.compose import ColumnTransformer
from sklearn.pipeline import Pipeline
from sklearn.model_selection import StratifiedKFold, cross_validate, cross_val_predict, GridSear[illegible]
from sklearn.metrics import r2_score, mean_squared_error, mean_absolute_error

from sklearn.svm import SVR
from sklearn.ensemble import RandomForestRegressor, GradientBoostingRegressor
from sklearn.linear_model import Ridge, ElasticNet, Lasso
from sklearn.neighbors import KNeighborsRegressor

warnings.filterwarnings("ignore")
plt.style.use('seaborn-v0_8-darkgrid')
sns.set_palette("husl")

# SHAP import
try:
    import shap
    HAS_SHAP = True
except ImportError:
    HAS_SHAP = False
    print("Warning: SHAP not installed. Install with: pip install shap")

# Configuration
DATA_PATH = "dataset.csv"
OUTDIR = Path("ML-OUTPUTS")
OUTDIR.mkdir(exist_ok=True, parents=True)
RANDOM_STATE = 42
N_FOLDS = 4
STRATIFY_THRESHOLD = 60

print("="*80)
print("ULTRA-COMPREHENSIVE SOLVENT RETENTION ANALYSIS")
print("="*80)
print(f"\nGenerating 30-50 detailed visualizations...")
print(f"Output directory: {OUTDIR}")
```

```
# Load data
data = pd.read_csv(DATA_PATH, encoding='utf-8-sig')
print(f"\nDataset: {data.shape[0]} samples, {data.shape[1]} columns")

# Feature mapping
feature_mapping = {
    'Polymer Mw': 'Mw',
    'Solvent Pvap': 'Pvap',
    'Solvent HSP': 'δT',
    'Predicted solubility': 'Spred',
    'Chi': 'χ',
    'Ratio': 'r'
}
feature_mapping_rev = {v: k for k, v in feature_mapping.items()}
target_col = "Solvent retention"
y_all = data[target_col].values

# Stratification
y_stratify = (y_all >= STRATIFY_THRESHOLD).astype(int)
cv = StratifiedKFold(n_splits=N_FOLDS, shuffle=True, random_state=RANDOM_STATE)

def create_preprocessor(numeric_features):
    return ColumnTransformer(
        transformers=[
            ("num", StandardScaler(), numeric_features)
        ],
        remainder="drop"
    )

# ============================================================================
# AUTOMATIC FEATURE SELECTION VIA LEAVE-ONE-OUT ANALYSIS
# ============================================================================

print("\n" + "="*80)
print("AUTOMATIC FEATURE SELECTION (Leave-One-Out R²)")
print("="*80)

def evaluate_feature_set_quick(features_to_use, actual_features_list, data, y, cv, y_stratify):
    """Quick evaluation of a feature set using SVR."""
    X_temp = data[actual_features_list].copy()
    preprocessor_temp = create_preprocessor(actual_features_list)

    model_temp = Pipeline([
        ('preprocessor', preprocessor_temp),
        ('regressor', SVR(kernel='rbf', C=100, epsilon=0.05, gamma=0.5))
    ])

    cv_results = cross_validate(
        model_temp, X_temp, y,
        cv=cv.split(X_temp, y_stratify),
        scoring='r2',
        n_jobs=-1
    )

    return cv_results['test_score'].mean()
```

```
# Start with all 6 features
six_features = ['Mw', 'Pvap', 'δT', 'Spred', 'χ', 'r']
actual_six_features = [feature_mapping_rev[f] for f in six_features]

print(f"\nStarting with 6 features: {six_features}")

# ============================================================================
# Find best 5-feature set (leave-one-out from 6 features)
# ============================================================================

print("\n" + "-"*60)
print("STEP 1: Finding best 5-feature set (leave-one-out)")
print("-"*60)

loo_results_5 = []

for feat_to_remove in six_features:
    remaining_5 = [f for f in six_features if f != feat_to_remove]
    actual_remaining_5 = [feature_mapping_rev[f] for f in remaining_5]

    print(f"\nTesting 5-feature set (removed {feat_to_remove}): {remaining_5}")

    r2_cv_score = evaluate_feature_set_quick(
        remaining_5, actual_remaining_5, data, y_all, cv, y_stratify
    )

    loo_results_5.append({
        'removed_feature': feat_to_remove,
        'remaining_features': remaining_5,
        'r2_mean': r2_cv_score
    })

    print(f"  R² = {r2_cv_score:.4f}")

# Find best 5-feature set
loo_df_5 = pd.DataFrame(loo_results_5).sort_values('r2_mean', ascending=False)
loo_df_5.reset_index(drop=True, inplace=True)
best_5_idx = loo_df_5['r2_mean'].idxmax()
best_removed_for_5 = loo_df_5.loc[best_5_idx, 'removed_feature']
five_features = loo_df_5.loc[best_5_idx, 'remaining_features']
best_r2_5 = loo_df_5.loc[best_5_idx, 'r2_mean']

print("\n" + "="*60)
print(f"BEST 5-FEATURE SET (R² = {best_r2_5:.4f})")
print(f"  Removed: {best_removed_for_5}")
print(f"  Kept: {five_features}")
print("="*60)

actual_five_features = [feature_mapping_rev[f] for f in five_features]

# ============================================================================
# Find best 4-feature set (leave-one-out from best 5 features)
# ============================================================================

print("\n" + "-"*60)
```

```python
print("STEP 2: Finding best 4-feature set (leave-one-out from best 5)")
print("-"*60)

loo_results_4 = []

for feat_to_remove in five_features:
    remaining_4 = [f for f in five_features if f != feat_to_remove]
    actual_remaining_4 = [feature_mapping_rev[f] for f in remaining_4]

    print(f"\nTesting 4-feature set (removed {feat_to_remove}): {remaining_4}")

    r2_cv_score = evaluate_feature_set_quick(
        remaining_4, actual_remaining_4, data, y_all, cv, y_stratify
    )

    loo_results_4.append({
        'removed_feature': feat_to_remove,
        'remaining_features': remaining_4,
        'r2_mean': r2_cv_score
    })

    print(f"  R² = {r2_cv_score:.4f}")

# Find best 4-feature set
loo_df_4 = pd.DataFrame(loo_results_4).sort_values('r2_mean', ascending=False)
loo_df_4.reset_index(drop=True, inplace=True)
best_4_idx = loo_df_4['r2_mean'].idxmax()
best_removed_for_4 = loo_df_4.loc[best_4_idx, 'removed_feature']
four_features = loo_df_4.loc[best_4_idx, 'remaining_features']
best_r2_4 = loo_df_4.loc[best_4_idx, 'r2_mean']

print("\n" + "="*60)
print(f"BEST 4-FEATURE SET (R² = {best_r2_4:.4f})")
print(f"  Removed from 5: {best_removed_for_4}")
print(f"  Kept: {four_features}")
print("="*60)

actual_four_features = [feature_mapping_rev[f] for f in four_features]

# ============================================================================
# Summary of feature selection
# ============================================================================

print("\n" + "="*80)
print("FEATURE SELECTION SUMMARY")
print("="*80)
print(f"6-feature set: {six_features} (R² baseline)")
print(f"5-feature set: {five_features} (R² = {best_r2_5:.4f}) [removed: {best_removed_for_5}]")
print(f"4-feature set: {four_features} (R² = {best_r2_4:.4f}) [removed: {best_removed_for_4}]")

# Visualization of leave-one-out results
fig, axes = plt.subplots(1, 2, figsize=(14, 5))

# 5-feature selection
ax = axes[0]
colors_5 = ['green' if i == best_5_idx else 'gray' for i in range(len(loo_df_5))]
```

```
ax.barh(range(len(loo_df_5)), loo_df_5['r2_mean'], color=colors_5, alpha=0.7)
ax.set_yticks(range(len(loo_df_5)))
ax.set_yticklabels([f"Remove {r}" for r in loo_df_5['removed_feature']])
ax.set_xlabel('Cross-Validation R²', fontsize=11, fontweight='bold')
ax.set_title(f'Leave-One-Out: 5-Feature Selection\nBest: Remove {best_removed_for_5} (R²={best_r
            fontsize=12, fontweight='bold')
ax.grid(axis='x', alpha=0.3)
for i, (idx, row) in enumerate(loo_df_5.iterrows()):
    ax.text(row['r2_mean'] + 0.001, i, f"{row['r2_mean']:.4f}",
           va='center', fontsize=9)

# 4-feature selection
ax = axes[1]
colors_4 = ['green' if i == best_4_idx else 'gray' for i in range(len(loo_df_4))]
ax.barh(range(len(loo_df_4)), loo_df_4['r2_mean'], color=colors_4, alpha=0.7)
ax.set_yticks(range(len(loo_df_4)))
ax.set_yticklabels([f"Remove {r}" for r in loo_df_4['removed_feature']])
ax.set_xlabel('Cross-Validation R²', fontsize=11, fontweight='bold')
ax.set_title(f'Leave-One-Out: 4-Feature Selection\nBest: Remove {best_removed_for_4} (R²={best_r
            fontsize=12, fontweight='bold')
ax.grid(axis='x', alpha=0.3)
for i, (idx, row) in enumerate(loo_df_4.iterrows()):
    ax.text(row['r2_mean'] + 0.001, i, f"{row['r2_mean']:.4f}",
           va='center', fontsize=9)

plt.tight_layout()
plt.savefig(OUTDIR / "fig00_feature_selection_loo.png", dpi=300, bbox_inches='tight')
plt.close()
print(f"\nFigure 0: Feature selection visualization saved")

# Define feature sets for rest of analysis
feature_sets = {
    '6-feature': six_features,
    '5-feature': five_features,
    '4-feature': four_features
}

actual_six_features = [feature_mapping_rev[f] for f in six_features]
actual_five_features = [feature_mapping_rev[f] for f in five_features]
actual_four_features = [feature_mapping_rev[f] for f in four_features]

target_col = "Solvent retention"

X_all = data[actual_six_features].copy()
y_all = data[target_col].values

# Stratification
y_stratify = (y_all >= STRATIFY_THRESHOLD).astype(int)
cv = StratifiedKFold(n_splits=N_FOLDS, shuffle=True, random_state=RANDOM_STATE)

print(f"\nFeature sets: {list(feature_sets.keys())}")
print(f"Cross-validation: {N_FOLDS}-fold Stratified at {STRATIFY_THRESHOLD}%")

# ==============================================================================
# SECTION 1: DETAILED CORRELATION ANALYSIS
# ==============================================================================
```

```
print("\n" + "="*80)
print("SECTION 1: DETAILED CORRELATION & LINEAR ANALYSIS")
print("="*80)

fig_num = 1

# Pearson vs Spearman comparison
numeric_features_actual = actual_six_features

correlations = []
for feat in numeric_features_actual:
    pearson_r, pearson_p = stats.pearsonr(data[feat], y_all)
    spearman_r, spearman_p = stats.spearmanr(data[feat], y_all)
    correlations.append({
        'Feature': feature_mapping[feat],
        'Feature_Full': feat,
        'Pearson_r': pearson_r,
        'Pearson_p': pearson_p,
        'Spearman_rho': spearman_r,
        'Spearman_p': spearman_p,
        'Abs_Pearson': abs(pearson_r),
        'Abs_Spearman': abs(spearman_r)
    })

corr_df = pd.DataFrame(correlations).sort_values('Abs_Spearman', ascending=False)
corr_df.to_csv(OUTDIR / "correlations_detailed.csv", index=False)

# Pearson correlation plot
fig, ax = plt.subplots(figsize=(8, 6))

colors_p = ['red' if p < 0.05 else 'gray' for p in corr_df['Pearson_p']]
ax.barh(corr_df['Feature'], corr_df['Pearson_r'], color=colors_p, alpha=0.7)
ax.axvline(0, color='black', linewidth=1)
ax.set_xlabel('Pearson Correlation (r)', fontsize=12, fontweight='bold')
ax.set_title('Pearson Correlation with Solvent Retention\n(Red = p < 0.05)',
             fontsize=13, fontweight='bold')
ax.grid(axis='x', alpha=0.3)

for i, (feat, val, p) in enumerate(
        zip(corr_df['Feature'], corr_df['Pearson_r'], corr_df['Pearson_p'])):
    ax.text(val + 0.02 if val > 0 else val - 0.02, i,
            f'{val:.3f}{"*" if p < 0.05 else ""}',
            va='center',
            ha='left' if val > 0 else 'right',
            fontsize=9)

plt.tight_layout()
plt.savefig(OUTDIR / f"fig{fig_num:02d}_correlation_pearson.png",
            dpi=300, bbox_inches='tight')
plt.close()
print(f"Figure {fig_num}: Pearson correlation")
fig_num += 1

# Spearman correlation plot
fig, ax = plt.subplots(figsize=(8, 6))
```

```
colors_s = ['red' if p < 0.05 else 'gray' for p in corr_df['Spearman_p']]
ax.barh(corr_df['Feature'], corr_df['Spearman_rho'], color=colors_s, alpha=0.7)
ax.axvline(0, color='black', linewidth=1)
ax.set_xlabel('Spearman Correlation (ρ)', fontsize=12, fontweight='bold')
ax.set_title('Spearman Correlation with Solvent Retention\n(Red = p < 0.05)',
             fontsize=13, fontweight='bold')
ax.grid(axis='x', alpha=0.3)

for i, (feat, val, p) in enumerate(
        zip(corr_df['Feature'], corr_df['Spearman_rho'], corr_df['Spearman_p'])):
    ax.text(val + 0.02 if val > 0 else val - 0.02, i,
            f'{val:.3f}{"*" if p < 0.05 else ""}',
            va='center',
            ha='left' if val > 0 else 'right',
            fontsize=9)

plt.tight_layout()
plt.savefig(OUTDIR / f"fig{fig_num:02d}_correlation_spearman.png",
            dpi=300, bbox_inches='tight')
plt.close()
print(f"Figure {fig_num}: Spearman correlation")
fig_num += 1

# Pearson vs Spearman comparison
fig, ax = plt.subplots(figsize=(8, 6))

x = np.arange(len(corr_df))
width = 0.35
ax.barh(x - width/2, corr_df['Pearson_r'], width, label='Pearson', alpha=0.8)
ax.barh(x + width/2, corr_df['Spearman_rho'], width, label='Spearman', alpha=0.8)

ax.set_yticks(x)
ax.set_yticklabels(corr_df['Feature'])
ax.axvline(0, color='black', linewidth=1)
ax.set_xlabel('Correlation Coefficient', fontsize=12, fontweight='bold')
ax.set_title('Pearson vs Spearman Correlation with Solvent Retention',
             fontsize=13, fontweight='bold')
ax.legend(fontsize=10)
ax.grid(axis='x', alpha=0.3)

plt.tight_layout()
plt.savefig(OUTDIR / f"fig{fig_num:02d}_correlation_pearson_vs_spearman.png",
            dpi=300, bbox_inches='tight')
plt.close()
print(f"Figure {fig_num}: Pearson vs Spearman comparison")
fig_num += 1

# Individual scatter plots with linear regression
n_vars = len(numeric_features_actual)
fig, axes = plt.subplots(2, 3, figsize=(18, 12))
axes = axes.flatten()

for idx, feat in enumerate(numeric_features_actual):
    ax = axes[idx]
    x_data = data[feat].values
```

```python
        y_data = y_all

        ax.scatter(x_data, y_data, s=80, alpha=0.6, edgecolors='black', linewidth=0.5)

        # Linear regression
        slope, intercept, r_value, p_value, std_err = stats.linregress(x_data, y_data)
        x_line = np.linspace(x_data.min(), x_data.max(), 100)
        y_line = slope * x_line + intercept
        ax.plot(x_line, y_line, 'r-', linewidth=2, label=f'y={slope:.2e}x+{intercept:.2f}')

        pearson_r = corr_df[corr_df['Feature_Full'] == feat]['Pearson_r'].values[0]
        spearman_r = corr_df[corr_df['Feature_Full'] == feat]['Spearman_rho'].values[0]

        ax.set_xlabel(feature_mapping[feat], fontsize=11, fontweight='bold')
        ax.set_ylabel('Solvent Retention (%)', fontsize=11, fontweight='bold')
        ax.set_title(f'{feature_mapping[feat]}\nPearson r={pearson_r:.3f}, Spearman ρ={spearman_r:.3
                    fontsize=11, fontweight='bold')
        ax.legend(fontsize=8)
        ax.grid(True, alpha=0.3)

    plt.tight_layout()
    plt.savefig(OUTDIR / f"fig{fig_num:02d}_individual_variable_analysis.png", dpi=300, bbox_inches=
    plt.close()
    print(f"Figure {fig_num}: Individual variable linear analysis")
    fig_num += 1

    # Correlation matrix heatmap (Pearson)
    corr_matrix = data[numeric_features_actual + [target_col]].corr(method='pearson')
    fig, ax = plt.subplots(figsize=(10, 8))
    sns.heatmap(corr_matrix, annot=True, fmt='.3f', cmap='RdBu_r', center=0,
                vmin=-1, vmax=1, square=True, linewidths=0.5,
                cbar_kws={'label': 'Pearson Correlation'}, ax=ax)
    labels = [feature_mapping.get(col, col) for col in corr_matrix.columns]
    ax.set_xticklabels(labels, rotation=45, ha='right')
    ax.set_yticklabels(labels, rotation=0)
    ax.set_title('Pearson Correlation Matrix\n(Including Target Variable)',
                fontsize=14, fontweight='bold')
    plt.tight_layout()
    plt.savefig(OUTDIR / f"fig{fig_num:02d}_correlation_matrix_pearson.png", dpi=300, bbox_inches='t
    plt.close()
    print(f"Figure {fig_num}: Pearson correlation matrix")
    fig_num += 1

    # Correlation matrix heatmap (Spearman)
    corr_matrix_spearman = data[numeric_features_actual + [target_col]].corr(method='spearman')
    fig, ax = plt.subplots(figsize=(10, 8))
    sns.heatmap(corr_matrix_spearman, annot=True, fmt='.3f', cmap='RdBu_r', center=0,
                vmin=-1, vmax=1, square=True, linewidths=0.5,
                cbar_kws={'label': 'Spearman Correlation'}, ax=ax)
    labels = [feature_mapping.get(col, col) for col in corr_matrix_spearman.columns]
    ax.set_xticklabels(labels, rotation=45, ha='right')
    ax.set_yticklabels(labels, rotation=0)
    ax.set_title('Spearman Correlation Matrix\n(Including Target Variable)',
                fontsize=14, fontweight='bold')
    plt.tight_layout()
    plt.savefig(OUTDIR / f"fig{fig_num:02d}_correlation_matrix_spearman.png", dpi=300, bbox_inches='
```

```python
plt.close()
print(f"Figure {fig_num}: Spearman correlation matrix")
fig_num += 1

# ============================================================================
# SECTION 2: MODEL TRAINING WITH EXTENSIVE HYPERPARAMETER TUNING
# ============================================================================

print("\n" + "="*80)
print("SECTION 2: MODEL TRAINING & HYPERPARAMETER OPTIMIZATION")
print("="*80)

# Hyperparameter grids
svr_param_grids = {
    '6-feature': {
        'C': [50, 60, 70, 80, 90, 100],
        'epsilon': [0.01, 0.03, 0.05, 0.07, 0.1],
        'gamma': [0.1, 0.2, 0.3, 0.4, 0.5]
    },
    '5-feature': {
        'C': [60, 70, 80, 90, 100, 110],
        'epsilon': [0.01, 0.02, 0.03, 0.05, 0.07],
        'gamma': [0.3, 0.4, 0.5, 0.55, 0.6, 0.7]
    },
    '4-feature': {
        'C': [80, 90, 100, 110, 120],
        'epsilon': [0.01, 0.02, 0.03, 0.05],
        'gamma': [0.4, 0.5, 0.55, 0.6, 0.7]
    }
}

rf_param_grid = {
    'n_estimators': [100, 200, 300, 500],
    'max_depth': [3, 5, 7, 10, 15, None],
    'min_samples_split': [2, 5, 10],
    'min_samples_leaf': [1, 2, 4],
    'max_features': ['sqrt', 'log2', 0.5]
}

gbm_param_grid = {
    'n_estimators': [100, 200, 300, 500],
    'learning_rate': [0.01, 0.03, 0.05, 0.1, 0.15],
    'max_depth': [2, 3, 4, 5],
    'min_samples_split': [2, 5, 10],
    'min_samples_leaf': [1, 2, 4],
    'subsample': [0.8, 0.9, 1.0]
}

ridge_param_grid = {
    'alpha': [0.001, 0.01, 0.1, 0.5, 1.0, 5.0, 10.0, 50.0, 100.0]
}

elasticnet_param_grid = {
    'alpha': [0.001, 0.01, 0.1, 0.5, 1.0, 5.0, 10.0],
    'l1_ratio': [0.1, 0.3, 0.5, 0.7, 0.9]
}
```

```python
knn_param_grid = {
    'n_neighbors': [3, 5, 7, 9, 11, 15],
    'weights': ['uniform', 'distance'],
    'p': [1, 2],
    'metric': ['euclidean', 'manhattan']
}

def make_ohe():
    try:
        return OneHotEncoder(handle_unknown="ignore", sparse_output=False)
    except TypeError:
        return OneHotEncoder(handle_unknown="ignore", sparse=False)

def create_preprocessor(numeric_features):
    return ColumnTransformer(
        transformers=[
            ("num", StandardScaler(), numeric_features)
        ],
        remainder="drop"
    )

def evaluate_model_detailed(pipeline, X, y, y_stratify, cv, model_name, feature_set_name):
    """Evaluate model with detailed metrics."""
    scoring = {
        'r2': 'r2',
        'neg_mae': 'neg_mean_absolute_error',
        'neg_rmse': 'neg_root_mean_squared_error',
        'neg_mse': 'neg_mean_squared_error'
    }

    cv_results = cross_validate(
        pipeline, X, y,
        cv=cv.split(X, y_stratify),
        scoring=scoring,
        n_jobs=-1,
        return_train_score=True
    )

    y_pred = cross_val_predict(pipeline, X, y, cv=cv.split(X, y_stratify), n_jobs=-1)
    residuals = y - y_pred

    mape = np.mean(np.abs((y - y_pred) / y)) * 100
    max_error = np.max(np.abs(residuals))

    results = {
        'model': model_name,
        'feature_set': feature_set_name,
        'train_r2_mean': cv_results['train_r2'].mean(),
        'train_r2_std': cv_results['train_r2'].std(),
        'test_r2_mean': cv_results['test_r2'].mean(),
        'test_r2_std': cv_results['test_r2'].std(),
        'test_mae_mean': -cv_results['test_neg_mae'].mean(),
        'test_mae_std': cv_results['test_neg_mae'].std(),
        'test_rmse_mean': -cv_results['test_neg_rmse'].mean(),
        'test_rmse_std': cv_results['test_neg_rmse'].std(),
```

```
        'test_mse_mean': -cv_results['test_neg_mse'].mean(),
        'mape': mape,
        'max_error': max_error,
        'predictions': y_pred,
        'residuals': residuals
    }

    return results

def grid_search_model_detailed(base_model, param_grid, preprocessor, X, y, y_stratify, cv):
    """Perform grid search and return detailed results."""
    pipeline = Pipeline([
        ('preprocessor', preprocessor),
        ('regressor', base_model)
    ])

    param_grid_pipeline = {f'regressor__{k}': v for k, v in param_grid.items()}

    grid_search = GridSearchCV(
        pipeline,
        param_grid_pipeline,
        cv=cv.split(X, y_stratify),
        scoring='r2',
        n_jobs=-1,
        verbose=0,
        return_train_score=True
    )

    grid_search.fit(X, y)
    cv_results_df = pd.DataFrame(grid_search.cv_results_)

    return grid_search.best_estimator_, grid_search.best_params_, grid_search.best_score_, cv_re

# Train all models
all_results = []
best_models = {}
grid_search_results = {}

for feat_set_name, feat_list in feature_sets.items():
    print(f"\n{'='*80}")
    print(f"FEATURE SET: {feat_set_name}")
    print(f"{'='*80}")

    actual_features = [feature_mapping_rev[f] for f in feat_list]
    X_subset = data[actual_features].copy()
    preprocessor = create_preprocessor(actual_features)

    best_models[feat_set_name] = {}
    grid_search_results[feat_set_name] = {}

    # SVR
    print(f"\n1. SVR (RBF kernel)")
    svr_grid = svr_param_grids[feat_set_name]
    best_svr, best_svr_params, best_svr_score, svr_cv_results = grid_search_model_detailed(
        SVR(kernel='rbf'), svr_grid, preprocessor, X_subset, y_all, y_stratify, cv
    )
```

```
grid_search_results[feat_set_name]['SVR'] = svr_cv_results
print(f"   Best params: {best_svr_params}")
print(f"   Best CV R²: {best_svr_score:.4f}")

svr_results = evaluate_model_detailed(best_svr, X_subset, y_all, y_stratify, cv, 'SVR', feat_
all_results.append(svr_results)
best_models[feat_set_name]['SVR'] = best_svr
print(f"   Test R²: {svr_results['test_r2_mean']:.4f} ± {svr_results['test_r2_std']:.4f}")

# Random Forest
print(f"\n2. Random Forest")
best_rf, best_rf_params, best_rf_score, rf_cv_results = grid_search_model_detailed(
    RandomForestRegressor(random_state=RANDOM_STATE), rf_param_grid,
    preprocessor, X_subset, y_all, y_stratify, cv
)
grid_search_results[feat_set_name]['Random Forest'] = rf_cv_results
print(f"   Best params: {best_rf_params}")
print(f"   Best CV R²: {best_rf_score:.4f}")

rf_results = evaluate_model_detailed(best_rf, X_subset, y_all, y_stratify, cv, 'Random Forest
all_results.append(rf_results)
best_models[feat_set_name]['Random Forest'] = best_rf
print(f"   Test R²: {rf_results['test_r2_mean']:.4f} ± {rf_results['test_r2_std']:.4f}")

# Gradient Boosting
print(f"\n3. Gradient Boosting")
best_gbm, best_gbm_params, best_gbm_score, gbm_cv_results = grid_search_model_detailed(
    GradientBoostingRegressor(random_state=RANDOM_STATE), gbm_param_grid,
    preprocessor, X_subset, y_all, y_stratify, cv
)
grid_search_results[feat_set_name]['Gradient Boosting'] = gbm_cv_results
print(f"   Best params: {best_gbm_params}")
print(f"   Best CV R²: {best_gbm_score:.4f}")

gbm_results = evaluate_model_detailed(best_gbm, X_subset, y_all, y_stratify, cv, 'Gradient Bo
all_results.append(gbm_results)
best_models[feat_set_name]['Gradient Boosting'] = best_gbm
print(f"   Test R²: {gbm_results['test_r2_mean']:.4f} ± {gbm_results['test_r2_std']:.4f}")

# Ridge
print(f"\n4. Ridge Regression")
best_ridge, best_ridge_params, best_ridge_score, ridge_cv_results = grid_search_model_detaile
    Ridge(random_state=RANDOM_STATE), ridge_param_grid,
    preprocessor, X_subset, y_all, y_stratify, cv
)
grid_search_results[feat_set_name]['Ridge'] = ridge_cv_results
print(f"   Best params: {best_ridge_params}")
print(f"   Best CV R²: {best_ridge_score:.4f}")

ridge_results = evaluate_model_detailed(best_ridge, X_subset, y_all, y_stratify, cv, 'Ridge',
all_results.append(ridge_results)
best_models[feat_set_name]['Ridge'] = best_ridge
print(f"   Test R²: {ridge_results['test_r2_mean']:.4f} ± {ridge_results['test_r2_std']:.4f}"

# ElasticNet
print(f"\n5. ElasticNet")
```

```
    best_en, best_en_params, best_en_score, en_cv_results = grid_search_model_detailed(
        ElasticNet(random_state=RANDOM_STATE, max_iter=10000), elasticnet_param_grid,
        preprocessor, X_subset, y_all, y_stratify, cv
    )
    grid_search_results[feat_set_name]['ElasticNet'] = en_cv_results
    print(f"   Best params: {best_en_params}")
    print(f"   Best CV R²: {best_en_score:.4f}")

    en_results = evaluate_model_detailed(best_en, X_subset, y_all, y_stratify, cv, 'ElasticNet',
    all_results.append(en_results)
    best_models[feat_set_name]['ElasticNet'] = best_en
    print(f"   Test R²: {en_results['test_r2_mean']:.4f} ± {en_results['test_r2_std']:.4f}")

    # KNN
    print(f"\n6. K-Nearest Neighbors")
    best_knn, best_knn_params, best_knn_score, knn_cv_results = grid_search_model_detailed(
        KNeighborsRegressor(), knn_param_grid,
        preprocessor, X_subset, y_all, y_stratify, cv
    )
    grid_search_results[feat_set_name]['KNN'] = knn_cv_results
    print(f"   Best params: {best_knn_params}")
    print(f"   Best CV R²: {best_knn_score:.4f}")

    knn_results = evaluate_model_detailed(best_knn, X_subset, y_all, y_stratify, cv, 'KNN', feat_
    all_results.append(knn_results)
    best_models[feat_set_name]['KNN'] = best_knn
    print(f"   Test R²: {knn_results['test_r2_mean']:.4f} ± {knn_results['test_r2_std']:.4f}")

# Save results
results_df = pd.DataFrame([{k: v for k, v in r.items() if k not in ['predictions', 'residuals']}
                           for r in all_results])
results_df.to_csv(OUTDIR / "all_model_results_detailed.csv", index=False)

print("\n" + "="*80)
print("SECTION 3: COMPREHENSIVE VISUALIZATIONS")
print("="*80)

# ============================================================================
# SECTION 3.1: SIDE-BY-SIDE R² COMPARISONS
# ============================================================================

print("\nCreating side-by-side R² comparisons...")

# R² comparison heatmap
pivot_r2 = results_df.pivot(index='model', columns='feature_set', values='test_r2_mean')
pivot_r2 = pivot_r2[['6-feature', '5-feature', '4-feature']]

fig, ax = plt.subplots(figsize=(10, 8))
sns.heatmap(pivot_r2, annot=True, fmt='.4f', cmap='RdYlGn', vmin=0, vmax=1,
            cbar_kws={'label': 'Test R²'}, ax=ax, linewidths=0.5)
ax.set_title('Model Performance Comparison (Test R²)\nAcross Feature Sets',
            fontsize=14, fontweight='bold')
ax.set_xlabel('Feature Set', fontsize=12, fontweight='bold')
ax.set_ylabel('Model', fontsize=12, fontweight='bold')
plt.tight_layout()
plt.savefig(OUTDIR / f"fig{fig_num:02d}_r2_heatmap_comparison.png", dpi=300, bbox_inches='tight'
```

```python
plt.close()
print(f"Figure {fig_num}: R² heatmap comparison")
fig_num += 1

# Side-by-side bar plots for all metrics
fig, axes = plt.subplots(2, 2, figsize=(16, 12))

metrics_to_plot = [
    ('test_r2_mean', 'Test R²', 'higher is better'),
    ('test_rmse_mean', 'Test RMSE (%)', 'lower is better'),
    ('test_mae_mean', 'Test MAE (%)', 'lower is better'),
    ('mape', 'MAPE (%)', 'lower is better')
]

for idx, (metric, ylabel, note) in enumerate(metrics_to_plot):
    ax = axes[idx // 2, idx % 2]

    x = np.arange(len(results_df['model'].unique()))
    width = 0.25

    for i, feat_set in enumerate(['6-feature', '5-feature', '4-feature']):
        subset = results_df[results_df['feature_set'] == feat_set]
        offset = (i - 1) * width
        ax.bar(x + offset, subset[metric], width, label=feat_set, alpha=0.8)

    ax.set_xlabel('Model', fontsize=11, fontweight='bold')
    ax.set_ylabel(ylabel, fontsize=11, fontweight='bold')
    ax.set_title(f'{ylabel} Comparison\n({note})', fontsize=12, fontweight='bold')
    ax.set_xticks(x)
    ax.set_xticklabels(subset['model'], rotation=45, ha='right')
    ax.legend(fontsize=9)
    ax.grid(axis='y', alpha=0.3)

plt.tight_layout()
plt.savefig(OUTDIR / f"fig{fig_num:02d}_metrics_side_by_side.png", dpi=300, bbox_inches='tight')
plt.close()
print(f"Figure {fig_num}: Metrics side-by-side comparison")
fig_num += 1

# Individual model performance across feature sets
fig, axes = plt.subplots(2, 3, figsize=(18, 12))
axes = axes.flatten()

models = results_df['model'].unique()
for idx, model in enumerate(models):
    ax = axes[idx]
    subset = results_df[results_df['model'] == model]

    x = np.arange(3)
    r2_vals = [subset[subset['feature_set'] == fs]['test_r2_mean'].values[0]
               for fs in ['6-feature', '5-feature', '4-feature']]
    r2_errs = [subset[subset['feature_set'] == fs]['test_r2_std'].values[0]
               for fs in ['6-feature', '5-feature', '4-feature']]

    bars = ax.bar(x, r2_vals, yerr=r2_errs, capsize=5, alpha=0.7,
                  color=['#1f77b4', '#ff7f0e', '#2ca02c'])
```

```
    ax.set_xticks(x)
    ax.set_xticklabels(['6-feat', '5-feat', '4-feat'])
    ax.set_ylabel('Test R²', fontsize=11, fontweight='bold')
    ax.set_title(f'{model}', fontsize=12, fontweight='bold')
    ax.set_ylim([0, 1])
    ax.grid(axis='y', alpha=0.3)

    for i, (bar, val, err) in enumerate(zip(bars, r2_vals, r2_errs)):
        ax.text(bar.get_x() + bar.get_width()/2, val + err + 0.02,
               f'{val:.3f}', ha='center', va='bottom', fontsize=9)

plt.tight_layout()
plt.savefig(OUTDIR / f"fig{fig_num:02d}_model_performance_by_features.png", dpi=300, bbox_inches=
plt.close()
print(f"Figure {fig_num}: Individual model performance by feature set")
fig_num += 1

# ============================================================================
# SECTION 3.2: PARITY PLOTS FOR BEST MODELS
# ============================================================================

print("\nCreating parity plots for best models...")

polymers = data['Polymer'].values
polymer_types = np.unique(polymers)
colors = plt.cm.tab10(np.linspace(0, 1, len(polymer_types)))
color_map = dict(zip(polymer_types, colors))

# Best model parity plots (3 panels combined)
fig, axes = plt.subplots(1, 3, figsize=(18, 6))

for idx, feat_set in enumerate(['6-feature', '5-feature', '4-feature']):
    ax = axes[idx]

    subset = results_df[results_df['feature_set'] == feat_set]
    best_idx = subset['test_r2_mean'].idxmax()
    best_for_fs = all_results[best_idx]

    y_true = y_all
    y_pred = best_for_fs['predictions']
    r2 = best_for_fs['test_r2_mean']
    rmse = best_for_fs['test_rmse_mean']
    mae = best_for_fs['test_mae_mean']

    for polymer in polymer_types:
        mask = polymers == polymer
        ax.scatter(
            y_true[mask], y_pred[mask],
            s=120, alpha=0.7,
            color=color_map[polymer],
            label=polymer if idx == 0 else '',
            edgecolors='black', linewidth=1
        )

    lims = [
        min(y_true.min(), y_pred.min()) - 5,
```

```
        max(y_true.max(), y_pred.max()) + 5
    ]

    z = np.polyfit(y_true, y_pred, 1)
    p = np.poly1d(z)
    ax.plot(lims, p(lims), 'r-', lw=2, alpha=0.5)

    ax.set_xlabel('True Solvent Retention (%)', fontsize=12, fontweight='bold')
    ax.set_ylabel('Predicted Solvent Retention (%)', fontsize=12, fontweight='bold')
    ax.set_title(
        f'{feat_set}: {best_for_fs["model"]}\nR²={r2:.4f}, RMSE={rmse:.2f}%, MAE={mae:.2f}%',
        fontsize=12, fontweight='bold'
    )
    ax.grid(True, alpha=0.3)
    ax.set_xlim(lims)
    ax.set_ylim(lims)

    if idx == 0:
        ax.legend(loc='upper left', fontsize=8)

plt.tight_layout()
plt.savefig(OUTDIR / f"fig{fig_num:02d}_parity_best_models_combined.png",
            dpi=300, bbox_inches='tight')
plt.close()
print(f"Figure {fig_num}: Parity plots for best models (combined)")
fig_num += 1

# Individual parity plots for each feature set
for feat_set in ['6-feature', '5-feature', '4-feature']:
    subset = results_df[results_df['feature_set'] == feat_set]
    best_idx = subset['test_r2_mean'].idxmax()
    best_for_fs = all_results[best_idx]

    y_true = y_all
    y_pred = best_for_fs['predictions']
    r2 = best_for_fs['test_r2_mean']
    rmse = best_for_fs['test_rmse_mean']
    mae = best_for_fs['test_mae_mean']

    fig, ax = plt.subplots(figsize=(10, 8))

    for polymer in polymer_types:
        mask = polymers == polymer
        ax.scatter(
            y_true[mask], y_pred[mask],
            s=150, alpha=0.7,
            color=color_map[polymer], label=polymer,
            edgecolors='black', linewidth=1
        )

    lims = [
        min(y_true.min(), y_pred.min()) - 5,
        max(y_true.max(), y_pred.max()) + 5
    ]

    z = np.polyfit(y_true, y_pred, 1)
```

```
    p = np.poly1d(z)
    ax.plot(lims, p(lims), 'r-', lw=2, alpha=0.5)

    ax.set_xlabel('True Solvent Retention (%)', fontsize=13, fontweight='bold')
    ax.set_ylabel('Predicted Solvent Retention (%)', fontsize=13, fontweight='bold')
    ax.set_title(
        f'{feat_set}: {best_for_fs["model"]}\nR²={r2:.4f}, RMSE={rmse:.2f}%, MAE={mae:.2f}%',
        fontsize=14, fontweight='bold'
    )
    ax.grid(True, alpha=0.3)
    ax.set_xlim(lims)
    ax.set_ylim(lims)
    ax.legend(loc='upper left', fontsize=10)

    plt.tight_layout()
    plt.savefig(
        OUTDIR / f"fig{fig_num:02d}_parity_best_{feat_set.replace('-', '_')}.png",
        dpi=300, bbox_inches='tight'
    )
    plt.close()
    print(f"Figure {fig_num}: Parity plot for best model ({feat_set})")
    fig_num += 1

# Individual parity plots for ALL model-feature combinations
fig, axes = plt.subplots(6, 3, figsize=(18, 36))

result_idx = 0
for row, model in enumerate(models):
    for col, feat_set in enumerate(['6-feature', '5-feature', '4-feature']):
        ax = axes[row, col]

        result = all_results[result_idx]
        y_true = y_all
        y_pred = result['predictions']
        r2 = result['test_r2_mean']
        rmse = result['test_rmse_mean']
        mae = result['test_mae_mean']

        for polymer in polymer_types:
            mask = polymers == polymer
            ax.scatter(
                y_true[mask], y_pred[mask],
                s=80, alpha=0.6,
                color=color_map[polymer],
                edgecolors='black', linewidth=0.5
            )

        lims = [
            min(y_true.min(), y_pred.min()) - 5,
            max(y_true.max(), y_pred.max()) + 5
        ]

        z = np.polyfit(y_true, y_pred, 1)
        p = np.poly1d(z)
        ax.plot(lims, p(lims), 'r-', lw=1.5, alpha=0.5)
```

```
        ax.set_xlabel('True (%)', fontsize=10)
        ax.set_ylabel('Predicted (%)', fontsize=10)
        ax.set_title(
            f'{model} - {feat_set}\nR²={r2:.4f}, RMSE={rmse:.2f}%, MAE={mae:.2f}%',
            fontsize=10, fontweight='bold'
        )
        ax.grid(True, alpha=0.3)
        ax.set_xlim(lims)
        ax.set_ylim(lims)

        result_idx += 1

plt.tight_layout()
plt.savefig(
    OUTDIR / f"fig{fig_num:02d}_parity_all_combinations_combined.png",
    dpi=300, bbox_inches='tight'
)
plt.close()
print(f"Figure {fig_num}: Parity plots for all combinations (combined)")
fig_num += 1

# Individual 6-panel plots for each feature set
for feat_set in ['6-feature', '5-feature', '4-feature']:
    fig, axes = plt.subplots(2, 3, figsize=(18, 12))
    axes = axes.flatten()

    for idx, model in enumerate(models):
        ax = axes[idx]

        result = [
            r for r in all_results
            if r['model'] == model and r['feature_set'] == feat_set
        ][0]

        y_true = y_all
        y_pred = result['predictions']
        r2 = result['test_r2_mean']
        rmse = result['test_rmse_mean']
        mae = result['test_mae_mean']

        for polymer in polymer_types:
            mask = polymers == polymer
            ax.scatter(
                y_true[mask], y_pred[mask],
                s=100, alpha=0.7,
                color=color_map[polymer],
                edgecolors='black', linewidth=0.5,
                label=polymer if idx == 0 else ''
            )

        lims = [
            min(y_true.min(), y_pred.min()) - 5,
            max(y_true.max(), y_pred.max()) + 5
        ]

        z = np.polyfit(y_true, y_pred, 1)
```

```
        p = np.poly1d(z)
        ax.plot(lims, p(lims), 'r-', lw=2, alpha=0.5)

        ax.set_xlabel('True (%)', fontsize=11, fontweight='bold')
        ax.set_ylabel('Predicted (%)', fontsize=11, fontweight='bold')
        ax.set_title(
            f'{model}\nR²={r2:.4f}, RMSE={rmse:.2f}%, MAE={mae:.2f}%',
            fontsize=11, fontweight='bold'
        )
        ax.grid(True, alpha=0.3)
        ax.set_xlim(lims)
        ax.set_ylim(lims)

        if idx == 0:
            ax.legend(loc='upper left', fontsize=8)

    plt.suptitle(f'All Models - {feat_set}', fontsize=16, fontweight='bold')
    plt.tight_layout()
    plt.savefig(
        OUTDIR / f"fig{fig_num:02d}_parity_all_models_{feat_set.replace('-', '_')}.png",
        dpi=300, bbox_inches='tight'
    )
    plt.close()
    print(f"Figure {fig_num}: Parity plots for all models ({feat_set})")
    fig_num += 1

# ============================================================================
# SECTION 3.3: COMPLETE RESIDUAL ANALYSIS
# ============================================================================

print("\nCreating comprehensive residual analysis...")

# Residual analysis for best models (4 panels per feature set)
for feat_set in ['6-feature', '5-feature', '4-feature']:
    fig, axes = plt.subplots(2, 2, figsize=(14, 12))

    subset = results_df[results_df['feature_set'] == feat_set]
    best_idx = subset['test_r2_mean'].idxmax()
    best_result = all_results[best_idx]

    y_true = y_all
    y_pred = best_result['predictions']
    residuals = best_result['residuals']

    # Residuals vs Predicted
    ax = axes[0, 0]
    ax.scatter(y_pred, residuals, s=80, alpha=0.6, edgecolors='black', linewidth=0.5)
    ax.axhline(0, color='red', linestyle='--', linewidth=2)
    ax.set_xlabel('Predicted Values (%)', fontsize=11, fontweight='bold')
    ax.set_ylabel('Residuals (%)', fontsize=11, fontweight='bold')
    ax.set_title(f'Residuals vs Predicted\n{best_result["model"]} - {feat_set}',
                fontsize=12, fontweight='bold')
    ax.grid(True, alpha=0.3)

    std_resid = np.std(residuals)
    ax.axhline(2*std_resid, color='orange', linestyle=':', linewidth=1.5, alpha=0.7, label='±2σ'
```

```
    ax.axhline(-2*std_resid, color='orange', linestyle=':', linewidth=1.5, alpha=0.7)
    ax.legend()

    # Histogram of residuals
    ax = axes[0, 1]
    ax.hist(residuals, bins=15, edgecolor='black', alpha=0.7, color='skyblue')
    ax.axvline(0, color='red', linestyle='--', linewidth=2)
    ax.axvline(residuals.mean(), color='green', linestyle='-', linewidth=2,
              label=f'Mean={residuals.mean():.2f}')
    ax.set_xlabel('Residuals (%)', fontsize=11, fontweight='bold')
    ax.set_ylabel('Frequency', fontsize=11, fontweight='bold')
    ax.set_title(f'Residual Distribution\nStd={np.std(residuals):.2f}%, Skew={stats.skew(residua
                fontsize=12, fontweight='bold')
    ax.legend()
    ax.grid(True, alpha=0.3, axis='y')

    # Q-Q plot
    ax = axes[1, 0]
    stats.probplot(residuals, dist="norm", plot=ax)
    ax.set_title(f'Q-Q Plot (Normality Check)\n{best_result["model"]} - {feat_set}',
                fontsize=12, fontweight='bold')
    ax.grid(True, alpha=0.3)

    # Residuals by polymer
    ax = axes[1, 1]
    polymer_residuals = [residuals[polymers == p] for p in polymer_types]
    bp = ax.boxplot(polymer_residuals, labels=polymer_types, patch_artist=True, widths=0.6)
    for patch, color in zip(bp['boxes'], colors):
        patch.set_facecolor(color)
        patch.set_alpha(0.7)
    ax.axhline(0, color='red', linestyle='--', linewidth=2)
    ax.set_xlabel('Polymer Type', fontsize=11, fontweight='bold')
    ax.set_ylabel('Residuals (%)', fontsize=11, fontweight='bold')
    ax.set_title(f'Residuals by Polymer\n{best_result["model"]} - {feat_set}',
                fontsize=12, fontweight='bold')
    ax.grid(True, alpha=0.3, axis='y')

    plt.tight_layout()
    plt.savefig(OUTDIR / f"fig{fig_num:02d}_residual_analysis_{feat_set.replace('-', '_')}.png",
               dpi=300, bbox_inches='tight')
    plt.close()
    print(f"Figure {fig_num}: Residual analysis for {feat_set}")
    fig_num += 1

# Residual analysis for ALL models (combined overview)
fig, axes = plt.subplots(6, 3, figsize=(18, 36))

result_idx = 0
for row, model in enumerate(models):
    for col, feat_set in enumerate(['6-feature', '5-feature', '4-feature']):
        ax = axes[row, col]

        result = all_results[result_idx]
        residuals = result['residuals']
        y_pred = result['predictions']
```

```
        ax.scatter(y_pred, residuals, s=60, alpha=0.6, edgecolors='black', linewidth=0.5)
        ax.axhline(0, color='red', linestyle='--', linewidth=1.5)

        std_resid = np.std(residuals)
        ax.axhline(2*std_resid, color='orange', linestyle=':', linewidth=1, alpha=0.7)
        ax.axhline(-2*std_resid, color='orange', linestyle=':', linewidth=1, alpha=0.7)

        ax.set_xlabel('Predicted (%)', fontsize=9)
        ax.set_ylabel('Residuals (%)', fontsize=9)
        ax.set_title(f'{model} - {feat_set}\nStd={std_resid:.2f}%',
                    fontsize=10, fontweight='bold')
        ax.grid(True, alpha=0.3)

        result_idx += 1

plt.tight_layout()
plt.savefig(OUTDIR / f"fig{fig_num:02d}_residuals_all_models.png", dpi=300, bbox_inches='tight')
plt.close()
print(f"Figure {fig_num}: Residual plots for all models")
fig_num += 1

# Residual standard deviation comparison
fig, ax = plt.subplots(figsize=(12, 6))

x = np.arange(len(models))
width = 0.25

for i, feat_set in enumerate(['6-feature', '5-feature', '4-feature']):
    subset_results = [all_results[j] for j in range(len(all_results))
                     if all_results[j]['feature_set'] == feat_set]
    std_residuals = [np.std(r['residuals']) for r in subset_results]
    offset = (i - 1) * width
    ax.bar(x + offset, std_residuals, width, label=feat_set, alpha=0.8)

ax.set_xlabel('Model', fontsize=12, fontweight='bold')
ax.set_ylabel('Residual Standard Deviation (%)', fontsize=12, fontweight='bold')
ax.set_title('Residual Variability Comparison\n(Lower is better)', fontsize=14, fontweight='bold
ax.set_xticks(x)
ax.set_xticklabels(models, rotation=45, ha='right')
ax.legend(fontsize=10)
ax.grid(axis='y', alpha=0.3)

plt.tight_layout()
plt.savefig(OUTDIR / f"fig{fig_num:02d}_residual_std_comparison.png", dpi=300, bbox_inches='tigh
plt.close()
print(f"Figure {fig_num}: Residual standard deviation comparison")
fig_num += 1

# ============================================================================
# SECTION 3.4: AIC/BIC ANALYSIS FOR SVR
# ============================================================================

print("\nCreating AIC/BIC analysis for SVR models...")

def calculate_aic_bic_svr(y_true, y_pred, n_params):
    """Calculate AIC/BIC for SVR models."""
```

```
    n = len(y_true)
    mse = mean_squared_error(y_true, y_pred)

    aic = n * np.log(mse) + 2 * n_params
    bic = n * np.log(mse) + n_params * np.log(n)

    return aic, bic

# Calculate AIC/BIC for SVR models
svr_info_criteria = []

for feat_set in ['6-feature', '5-feature', '4-feature']:
    svr_result = [r for r in all_results if r['model'] == 'SVR' and r['feature_set'] == feat_set

    y_pred = svr_result['predictions']
    svr_model = best_models[feat_set]['SVR']
    n_support_vectors = len(svr_model.named_steps['regressor'].support_)

    # Effective parameters: support vectors + bias + hyperparameters
    n_params = n_support_vectors + 1 + 3

    aic, bic = calculate_aic_bic_svr(y_all, y_pred, n_params)

    svr_info_criteria.append({
        'feature_set': feat_set,
        'n_support_vectors': n_support_vectors,
        'n_params': n_params,
        'AIC': aic,
        'BIC': bic,
        'r2': svr_result['test_r2_mean'],
        'rmse': svr_result['test_rmse_mean']
    })

svr_ic_df = pd.DataFrame(svr_info_criteria)
svr_ic_df.to_csv(OUTDIR / "svr_information_criteria.csv", index=False)

# AIC/BIC comparison for SVR
fig, axes = plt.subplots(2, 2, figsize=(14, 10))

# AIC comparison
ax = axes[0, 0]
x = np.arange(3)
bars = ax.bar(x, svr_ic_df['AIC'], alpha=0.7, color=['#1f77b4', '#ff7f0e', '#2ca02c'])
ax.set_xticks(x)
ax.set_xticklabels(['6-feature', '5-feature', '4-feature'])
ax.set_ylabel('AIC', fontsize=12, fontweight='bold')
ax.set_title('Akaike Information Criterion (AIC)\nfor SVR Models\n(Lower is better)',
            fontsize=13, fontweight='bold')
ax.grid(axis='y', alpha=0.3)
for bar, val in zip(bars, svr_ic_df['AIC']):
    ax.text(bar.get_x() + bar.get_width()/2, val, f'{val:.1f}',
           ha='center', va='bottom', fontsize=10, fontweight='bold')

# BIC comparison
ax = axes[0, 1]
bars = ax.bar(x, svr_ic_df['BIC'], alpha=0.7, color=['#1f77b4', '#ff7f0e', '#2ca02c'])
```

```
ax.set_xticks(x)
ax.set_xticklabels(['6-feature', '5-feature', '4-feature'])
ax.set_ylabel('BIC', fontsize=12, fontweight='bold')
ax.set_title('Bayesian Information Criterion (BIC)\nfor SVR Models\n(Lower is better)',
             fontsize=13, fontweight='bold')
ax.grid(axis='y', alpha=0.3)
for bar, val in zip(bars, svr_ic_df['BIC']):
    ax.text(bar.get_x() + bar.get_width()/2, val, f'{val:.1f}',
           ha='center', va='bottom', fontsize=10, fontweight='bold')

# Support vectors
ax = axes[1, 0]
bars = ax.bar(x, svr_ic_df['n_support_vectors'], alpha=0.7, color=['#1f77b4', '#ff7f0e', '#2ca02[illegible]
ax.set_xticks(x)
ax.set_xticklabels(['6-feature', '5-feature', '4-feature'])
ax.set_ylabel('Number of Support Vectors', fontsize=12, fontweight='bold')
ax.set_title('SVR Model Complexity\n(Support Vectors)', fontsize=13, fontweight='bold')
ax.grid(axis='y', alpha=0.3)
for bar, val in zip(bars, svr_ic_df['n_support_vectors']):
    ax.text(bar.get_x() + bar.get_width()/2, val, f'{int(val)}',
           ha='center', va='bottom', fontsize=10, fontweight='bold')

# AIC vs R² tradeoff
ax = axes[1, 1]
colors_ic = ['#1f77b4', '#ff7f0e', '#2ca02c']
for i, (fs, aic, r2) in enumerate(zip(['6-feat', '5-feat', '4-feat'],
                                       svr_ic_df['AIC'], svr_ic_df['r2'])):
    ax.scatter(aic, r2, s=300, alpha=0.7, color=colors_ic[i], edgecolors='black', linewidth=2)
    ax.annotate(fs, (aic, r2), fontsize=11, fontweight='bold',
               ha='center', va='center')

ax.set_xlabel('AIC (Lower is better)', fontsize=12, fontweight='bold')
ax.set_ylabel('R² (Higher is better)', fontsize=12, fontweight='bold')
ax.set_title('SVR: Model Fit vs Complexity\nTradeoff', fontsize=13, fontweight='bold')
ax.grid(True, alpha=0.3)

ax.annotate('', xy=(ax.get_xlim()[0], ax.get_ylim()[1]),
           xytext=(ax.get_xlim()[1], ax.get_ylim()[0]),
           arrowprops=dict(arrowstyle='->', color='green', lw=2, alpha=0.3))
ax.text((ax.get_xlim()[0] + ax.get_xlim()[1])/2,
       (ax.get_ylim()[0] + ax.get_ylim()[1])/2,
       'Ideal\nRegion', fontsize=12, color='green', alpha=0.5,
       ha='center', va='center', fontweight='bold')

plt.tight_layout()
plt.savefig(OUTDIR / f"fig{fig_num:02d}_svr_aic_bic_analysis.png", dpi=300, bbox_inches='tight')
plt.close()
print(f"Figure {fig_num}: SVR AIC/BIC analysis")
fig_num += 1

# ============================================================================
# SECTION 3.5: HYPERPARAMETER TUNING VISUALIZATIONS
# ============================================================================

print("\nCreating hyperparameter tuning visualizations...")
```

```
# SVR hyperparameter heatmaps
for feat_set in ['6-feature', '5-feature', '4-feature']:
    svr_cv_results = grid_search_results[feat_set]['SVR']

    c_values = []
    eps_values = []
    gamma_values = []
    r2_scores = []

    for idx in range(len(svr_cv_results)):
        params = svr_cv_results.loc[idx, 'params']
        c_values.append(params['regressor__C'])
        eps_values.append(params['regressor__epsilon'])
        gamma_values.append(params['regressor__gamma'])
        r2_scores.append(svr_cv_results.loc[idx, 'mean_test_score'])

    fig, axes = plt.subplots(1, 3, figsize=(18, 5))

    # C vs epsilon
    ax = axes[0]
    pivot_data = pd.DataFrame({
        'C': c_values,
        'epsilon': eps_values,
        'R2': r2_scores
    }).pivot_table(values='R2', index='epsilon', columns='C', aggfunc='mean')

    sns.heatmap(pivot_data, annot=True, fmt='.3f', cmap='viridis', ax=ax,
                cbar_kws={'label': 'CV R²'})
    ax.set_title(f'SVR Hyperparameters: C vs ε\n{feat_set} (averaged over γ)',
                 fontsize=12, fontweight='bold')
    ax.set_xlabel('C', fontsize=11, fontweight='bold')
    ax.set_ylabel('ε (epsilon)', fontsize=11, fontweight='bold')

    # C vs gamma
    ax = axes[1]
    pivot_data = pd.DataFrame({
        'C': c_values,
        'gamma': gamma_values,
        'R2': r2_scores
    }).pivot_table(values='R2', index='gamma', columns='C', aggfunc='mean')

    sns.heatmap(pivot_data, annot=True, fmt='.3f', cmap='viridis', ax=ax,
                cbar_kws={'label': 'CV R²'})
    ax.set_title(f'SVR Hyperparameters: C vs γ\n{feat_set} (averaged over ε)',
                 fontsize=12, fontweight='bold')
    ax.set_xlabel('C', fontsize=11, fontweight='bold')
    ax.set_ylabel('γ (gamma)', fontsize=11, fontweight='bold')

    # epsilon vs gamma
    ax = axes[2]
    pivot_data = pd.DataFrame({
        'epsilon': eps_values,
        'gamma': gamma_values,
        'R2': r2_scores
    }).pivot_table(values='R2', index='gamma', columns='epsilon', aggfunc='mean')
```

```
    sns.heatmap(pivot_data, annot=True, fmt='.3f', cmap='viridis', ax=ax,
                cbar_kws={'label': 'CV R²'})
    ax.set_title(f'SVR Hyperparameters: ε vs γ\n{feat_set} (averaged over C)',
                 fontsize=12, fontweight='bold')
    ax.set_xlabel('ε (epsilon)', fontsize=11, fontweight='bold')
    ax.set_ylabel('γ (gamma)', fontsize=11, fontweight='bold')

    plt.tight_layout()
    plt.savefig(OUTDIR / f"fig{fig_num:02d}_svr_hyperparameters_{feat_set.replace('-', '_')}.png'
                dpi=300, bbox_inches='tight')
    plt.close()
    print(f"Figure {fig_num}: SVR hyperparameter heatmaps for {feat_set}")
    fig_num += 1

# ============================================================================
# SECTION 3.6: MODEL COMPARISON VISUALIZATIONS
# ============================================================================

print("\nCreating additional comparison visualizations...")

# Train vs Test R² comparison
fig, ax = plt.subplots(figsize=(12, 8))

x = np.arange(len(results_df))
width = 0.35

bars1 = ax.bar(x - width/2, results_df['train_r2_mean'], width,
              label='Train R²', alpha=0.8, color='skyblue')
bars2 = ax.bar(x + width/2, results_df['test_r2_mean'], width,
              label='Test R²', alpha=0.8, color='coral')

ax.set_xlabel('Model - Feature Set', fontsize=12, fontweight='bold')
ax.set_ylabel('R²', fontsize=12, fontweight='bold')
ax.set_title('Train vs Test R² Comparison\n(Detecting Overfitting)',
            fontsize=14, fontweight='bold')
ax.set_xticks(x)
labels = [f"{r['model']}\n{r['feature_set']}" for _, r in results_df.iterrows()]
ax.set_xticklabels(labels, rotation=45, ha='right', fontsize=8)
ax.legend(fontsize=11)
ax.grid(axis='y', alpha=0.3)
ax.set_ylim([0, 1])

# Mark significant overfitting
for i, (train_r2, test_r2) in enumerate(zip(results_df['train_r2_mean'], results_df['test_r2_mea
    gap = train_r2 - test_r2
    if gap > 0.1:
        ax.plot([i-width/2, i+width/2], [train_r2, test_r2], 'r-', linewidth=2, alpha=0.5)
        ax.text(i, (train_r2 + test_r2)/2, f'Δ={gap:.2f}',
               ha='center', fontsize=7, color='red', fontweight='bold')

plt.tight_layout()
plt.savefig(OUTDIR / f"fig{fig_num:02d}_train_vs_test_r2.png", dpi=300, bbox_inches='tight')
plt.close()
print(f"Figure {fig_num}: Train vs Test R² comparison")
fig_num += 1
```

```
# Error metrics comparison
fig, axes = plt.subplots(1, 3, figsize=(18, 6))

error_metrics = [
    ('test_mae_mean', 'Mean Absolute Error (MAE) [%]'),
    ('test_rmse_mean', 'Root Mean Squared Error (RMSE) [%]'),
    ('max_error', 'Maximum Absolute Error [%]')
]

for idx, (metric, title) in enumerate(error_metrics):
    ax = axes[idx]

    x = np.arange(len(models))
    width = 0.25

    for i, feat_set in enumerate(['6-feature', '5-feature', '4-feature']):
        subset = results_df[results_df['feature_set'] == feat_set]
        values = subset[metric].values
        offset = (i - 1) * width
        ax.bar(x + offset, values, width, label=feat_set, alpha=0.8)

    ax.set_xlabel('Model', fontsize=11, fontweight='bold')
    ax.set_ylabel(title, fontsize=11, fontweight='bold')
    ax.set_title(f'{title}\n(Lower is better)', fontsize=12, fontweight='bold')
    ax.set_xticks(x)
    ax.set_xticklabels(models, rotation=45, ha='right')
    ax.legend(fontsize=9)
    ax.grid(axis='y', alpha=0.3)

plt.tight_layout()
plt.savefig(OUTDIR / f"fig{fig_num:02d}_error_metrics_comparison.png", dpi=300, bbox_inches='tig
plt.close()
print(f"Figure {fig_num}: Error metrics comparison")
fig_num += 1

# ============================================================================
# EXHAUSTIVE SHAP & FEATURE IMPORTANCE ANALYSIS
# ============================================================================

if HAS_SHAP:
    print("\n" + "="*80)
    print("EXHAUSTIVE SHAP ANALYSIS")
    print("="*80)

    for feat_set_name, feat_list in feature_sets.items():
        print(f"\n{'='*60}")
        print(f"SHAP Analysis: {feat_set_name}")
        print(f"{'='*60}")

        actual_features = [feature_mapping_rev[f] for f in feat_list]
        X_shap = data[actual_features].copy()

        subset_results = results_df[results_df['feature_set'] == feat_set_name]
        best_model_name = subset_results.loc[subset_results['test_r2_mean'].idxmax(), 'model']
        best_model_pipeline = best_models[feat_set_name][best_model_name]
```

```python
print(f"Analyzing: {best_model_name}")

try:
    best_model_pipeline.fit(X_shap, y_all)
    X_transformed = best_model_pipeline.named_steps['preprocessor'].transform(X_shap)

    feature_names = []
    for name, trans, cols in best_model_pipeline.named_steps['preprocessor'].transformers
        if name == 'num':
            feature_names.extend([feature_mapping[c] for c in cols])
        elif 'ohe' in name:
            try:
                ohe_names = trans.get_feature_names_out(cols)
                feature_names.extend(ohe_names)
            except:
                n_cats = X_transformed.shape[1] - len(feat_list)
                feature_names.extend([f'{cols[0]}_{i}' for i in range(n_cats)])

    model = best_model_pipeline.named_steps['regressor']

    if best_model_name in ['Random Forest', 'Gradient Boosting']:
        print("  Using TreeExplainer...")
        explainer = shap.TreeExplainer(model)
        shap_values = explainer.shap_values(X_transformed)
        base_value = explainer.expected_value

    else:
        print("  Using KernelExplainer (sampling 100 background)...")
        background = shap.sample(X_transformed, 100)
        explainer = shap.KernelExplainer(model.predict, background)
        shap_values = explainer.shap_values(X_transformed)
        base_value = explainer.expected_value

    if np.isscalar(base_value):
        base_values_array = np.full(X_transformed.shape[0], base_value)
    else:
        base_values_array = base_value

    shap_explanation = shap.Explanation(
        values=shap_values,
        base_values=base_values_array,
        data=X_transformed,
        feature_names=feature_names
    )

    # Summary plot (beeswarm)
    fig = plt.figure(figsize=(12, 8))
    shap.summary_plot(shap_explanation, show=False, max_display=20)
    plt.title(f'SHAP Summary Plot\n{feat_set_name}: {best_model_name}',
             fontsize=14, fontweight='bold')
    plt.tight_layout()
    plt.savefig(OUTDIR / f"fig{fig_num:02d}_shap_summary_{feat_set_name.replace('-', '_'
               dpi=300, bbox_inches='tight')
    plt.close()
    print(f"Figure {fig_num}: SHAP summary plot")
    fig_num += 1
```

```
# Bar plot of mean |SHAP|
fig = plt.figure(figsize=(10, 6))
shap.plots.bar(shap_explanation, show=False, max_display=20)
plt.title(f'SHAP Feature Importance (mean |SHAP|)\n{feat_set_name}: {best_model_name}
         fontsize=14, fontweight='bold')
plt.tight_layout()
plt.savefig(OUTDIR / f"fig{fig_num:02d}_shap_bar_{feat_set_name.replace('-', '_')}.pn
           dpi=300, bbox_inches='tight')
plt.close()
print(f"Figure {fig_num}: SHAP bar plot")
fig_num += 1

# Dependence plots for top features
shap_importance = np.abs(shap_explanation.values).mean(axis=0)
top_features_idx = np.argsort(shap_importance)[-6:][::-1]

n_top = min(6, len(top_features_idx))
fig, axes = plt.subplots(2, 3, figsize=(18, 12))
axes = axes.flatten()

for idx, feat_idx in enumerate(top_features_idx[:n_top]):
    ax = axes[idx]
    shap.dependence_plot(
        feat_idx,
        shap_explanation.values,
        X_transformed,
        feature_names=feature_names,
        show=False,
        ax=ax
    )
    ax.set_title(f'{feature_names[feat_idx]}\nSHAP Dependence',
               fontsize=11, fontweight='bold')

for idx in range(n_top, 6):
    axes[idx].axis('off')

plt.suptitle(f'SHAP Dependence Plots (Top Features)\n{feat_set_name}: {best_model_nam
            fontsize=14, fontweight='bold')
plt.tight_layout()
plt.savefig(OUTDIR / f"fig{fig_num:02d}_shap_dependence_{feat_set_name.replace('-',
           dpi=300, bbox_inches='tight')
plt.close()
print(f"Figure {fig_num}: SHAP dependence plots")
fig_num += 1

# Waterfall plots for representative samples
sample_indices = [0, len(y_all)//4, len(y_all)//2, 3*len(y_all)//4, len(y_all)-1]

for idx, sample_idx in enumerate(sample_indices):
    fig = plt.figure(figsize=(16, 6))
    shap.plots.waterfall(shap_explanation[sample_idx], max_display=10, show=False)
    plt.title(f'SHAP Waterfall Plot - Sample {sample_idx}\n{feat_set_name}: {best_mod
             fontsize=14, fontweight='bold', pad=15)
    plt.tight_layout()
    plt.savefig(OUTDIR / f"fig{fig_num:02d}_shap_waterfall_{feat_set_name.replace('-
```

```python
                dpi=300, bbox_inches='tight')
    plt.close()

print(f"Figures {fig_num}-{fig_num+4}: SHAP waterfall plots (5 samples)")
fig_num += 5

# Force plots for worst predictions
worst_pred_idx = np.argsort(np.abs(y_all - best_model_pipeline.predict(X_shap)))[-5:]

for idx, sample_idx in enumerate(worst_pred_idx):
    fig = plt.figure(figsize=(16, 4))
    shap.plots.force(shap_explanation[sample_idx], matplotlib=True, show=False)
    pred = best_model_pipeline.predict(X_shap.iloc[[sample_idx]])[0]
    error = y_all[sample_idx] - pred
    plt.title(f'SHAP Force Plot - Worst Prediction #{idx+1} (Sample {sample_idx})\n'
             f'{feat_set_name}: {best_model_name} | True={y_all[sample_idx]:.1f}%, Pr
             fontsize=13, fontweight='bold', pad=15)
    plt.tight_layout()
    plt.savefig(OUTDIR / f"fig{fig_num:02d}_shap_force_{feat_set_name.replace('-', '_
                dpi=300, bbox_inches='tight')
    plt.close()

print(f"Figures {fig_num}-{fig_num+4}: SHAP force plots (5 worst predictions)")
fig_num += 5

# Feature interaction heatmap
numeric_indices = list(range(len(feat_list)))
if len(numeric_indices) > 1:
    interaction_values = shap_explanation.values[:, numeric_indices]

    n_numeric = len(numeric_indices)
    interaction_matrix = np.zeros((n_numeric, n_numeric))

    for i in range(n_numeric):
        for j in range(i+1, n_numeric):
            interaction_matrix[i, j] = np.abs(np.corrcoef(
                interaction_values[:, i],
                interaction_values[:, j]
            )[0, 1])
            interaction_matrix[j, i] = interaction_matrix[i, j]

    fig, ax = plt.subplots(figsize=(10, 8))
    numeric_names = [feature_names[i] for i in numeric_indices]
    sns.heatmap(interaction_matrix, annot=True, fmt='.3f',
                xticklabels=numeric_names, yticklabels=numeric_names,
                cmap='YlOrRd', ax=ax, vmin=0, vmax=1)
    ax.set_title(f'SHAP Feature Interaction Strength\n{feat_set_name}: {best_model_na
                fontsize=14, fontweight='bold')
    plt.tight_layout()
    plt.savefig(OUTDIR / f"fig{fig_num:02d}_shap_interactions_{feat_set_name.replace
                dpi=300, bbox_inches='tight')
    plt.close()
    print(f"Figure {fig_num}: SHAP interaction heatmap")
    fig_num += 1

# Save SHAP values
```

```
            shap_df = pd.DataFrame(shap_explanation.values, columns=feature_names)
            shap_df['true_value'] = y_all
            shap_df['predicted_value'] = best_model_pipeline.predict(X_shap)
            shap_df.to_csv(OUTDIR / f"shap_values_{feat_set_name.replace('-', '_')}.csv", index=I
            print(f"Saved SHAP values to CSV")

        except Exception as e:
            print(f"Warning: SHAP analysis failed for {feat_set_name}: {e}")
            import traceback
            traceback.print_exc()

else:
    print("\nWarning: SHAP not available. Install with: pip install shap")

# ============================================================================
# PERMUTATION FEATURE IMPORTANCE
# ============================================================================

print("\n" + "="*80)
print("PERMUTATION FEATURE IMPORTANCE ANALYSIS")
print("="*80)

from sklearn.inspection import permutation_importance

for feat_set_name, feat_list in feature_sets.items():
    print(f"\n{feat_set_name}:")

    actual_features = [feature_mapping_rev[f] for f in feat_list]
    X_perm = data[actual_features].copy()

    subset_results = results_df[results_df['feature_set'] == feat_set_name]
    best_model_name = subset_results.loc[subset_results['test_r2_mean'].idxmax(), 'model']
    best_model_pipeline = best_models[feat_set_name][best_model_name]

    print(f"  Model: {best_model_name}")

    best_model_pipeline.fit(X_perm, y_all)

    perm_importance = permutation_importance(
        best_model_pipeline, X_perm, y_all,
        n_repeats=30, random_state=RANDOM_STATE, n_jobs=-1
    )

    feature_names_perm = [feature_mapping[f] for f in actual_features]

    perm_df = pd.DataFrame({
        'feature': feature_names_perm,
        'importance_mean': perm_importance.importances_mean[:len(feature_names_perm)],
        'importance_std': perm_importance.importances_std[:len(feature_names_perm)]
    }).sort_values('importance_mean', ascending=False)

    perm_df.to_csv(OUTDIR / f"permutation_importance_{feat_set_name.replace('-', '_')}.csv", inde

    fig, ax = plt.subplots(figsize=(10, 6))
    ax.barh(range(len(perm_df)), perm_df['importance_mean'],
           xerr=perm_df['importance_std'], capsize=5, alpha=0.7)
```

```
    ax.set_yticks(range(len(perm_df)))
    ax.set_yticklabels(perm_df['feature'])
    ax.set_xlabel('Permutation Importance (decrease in R²)', fontsize=11, fontweight='bold')
    ax.set_title(f'Permutation Feature Importance\n{feat_set_name}: {best_model_name}',
                fontsize=12, fontweight='bold')
    ax.grid(axis='x', alpha=0.3)
    plt.tight_layout()
    plt.savefig(OUTDIR / f"fig{fig_num:02d}_perm_importance_{feat_set_name.replace('-', '_')}.pn
               dpi=300, bbox_inches='tight')
    plt.close()
    print(f"Figure {fig_num}: Permutation importance")
    fig_num += 1

# ============================================================================
# PARTIAL DEPENDENCE PLOTS
# ============================================================================

print("\n" + "="*80)
print("PARTIAL DEPENDENCE PLOTS")
print("="*80)

from sklearn.inspection import PartialDependenceDisplay

for feat_set_name in ['6-feature']:
    actual_features = [feature_mapping_rev[f] for f in feature_sets[feat_set_name]]
    X_pdp = data[actual_features].copy()

    subset_results = results_df[results_df['feature_set'] == feat_set_name]
    best_model_name = subset_results.loc[subset_results['test_r2_mean'].idxmax(), 'model']
    best_model_pipeline = best_models[feat_set_name][best_model_name]

    print(f"\n{feat_set_name}: {best_model_name}")

    best_model_pipeline.fit(X_pdp, y_all)

    feature_indices = list(range(len(actual_features)))

    fig, ax = plt.subplots(figsize=(18, 10))
    display = PartialDependenceDisplay.from_estimator(
        best_model_pipeline, X_pdp, feature_indices,
        feature_names=[feature_mapping[f] for f in actual_features],
        n_cols=3, ax=ax, random_state=RANDOM_STATE
    )
    plt.suptitle(f'Partial Dependence Plots\n{feat_set_name}: {best_model_name}',
                fontsize=14, fontweight='bold')
    plt.tight_layout()
    plt.savefig(OUTDIR / f"fig{fig_num:02d}_partial_dependence.png", dpi=300, bbox_inches='tight
    plt.close()
    print(f"Figure {fig_num}: Partial dependence plots")
    fig_num += 1

# ============================================================================
# LEARNING CURVES
# ============================================================================

print("\n" + "="*80)
```

```
print("LEARNING CURVES ANALYSIS")
print("="*80)

from sklearn.model_selection import learning_curve

for feat_set_name in ['6-feature', '5-feature', '4-feature']:
    actual_features = [feature_mapping_rev[f] for f in feature_sets[feat_set_name]]
    X_lc = data[actual_features].copy()

    subset_results = results_df[results_df['feature_set'] == feat_set_name]
    best_model_name = subset_results.loc[subset_results['test_r2_mean'].idxmax(), 'model']
    best_model_pipeline = best_models[feat_set_name][best_model_name]

    print(f"\n{feat_set_name}: {best_model_name}")

    train_sizes = np.linspace(0.3, 1.0, 8)
    train_sizes_abs, train_scores, test_scores = learning_curve(
        best_model_pipeline, X_lc, y_all,
        train_sizes=train_sizes,
        cv=cv.split(X_lc, y_stratify),
        scoring='r2',
        n_jobs=-1,
        random_state=RANDOM_STATE
    )

    train_mean = train_scores.mean(axis=1)
    train_std = train_scores.std(axis=1)
    test_mean = test_scores.mean(axis=1)
    test_std = test_scores.std(axis=1)

    fig, ax = plt.subplots(figsize=(10, 6))
    ax.plot(train_sizes_abs, train_mean, 'o-', color='blue', label='Training score')
    ax.fill_between(train_sizes_abs, train_mean - train_std, train_mean + train_std,
                    alpha=0.2, color='blue')
    ax.plot(train_sizes_abs, test_mean, 'o-', color='red', label='Cross-validation score')
    ax.fill_between(train_sizes_abs, test_mean - test_std, test_mean + test_std,
                    alpha=0.2, color='red')

    ax.set_xlabel('Training Set Size', fontsize=11, fontweight='bold')
    ax.set_ylabel('R² Score', fontsize=11, fontweight='bold')
    ax.set_title(f'Learning Curve\n{feat_set_name}: {best_model_name}',
                 fontsize=12, fontweight='bold')
    ax.legend(loc='best', fontsize=10)
    ax.grid(True, alpha=0.3)
    plt.tight_layout()
    plt.savefig(OUTDIR / f"fig{fig_num:02d}_learning_curve_{feat_set_name.replace('-', '_')}.png'
                dpi=300, bbox_inches='tight')
    plt.close()
    print(f"Figure {fig_num}: Learning curve")
    fig_num += 1

# ============================================================================
# BOOTSTRAP CONFIDENCE INTERVALS
# ============================================================================

print("\n" + "="*80)
```

```
print("BOOTSTRAP CONFIDENCE INTERVALS")
print("="*80)

from sklearn.base import clone

n_bootstrap = 1000

for feat_set_name in ['6-feature']:
    actual_features = [feature_mapping_rev[f] for f in feature_sets[feat_set_name]]
    X_boot = data[actual_features].copy()

    subset_results = results_df[results_df['feature_set'] == feat_set_name]
    best_model_name = subset_results.loc[subset_results['test_r2_mean'].idxmax(), 'model']
    best_model_pipeline = best_models[feat_set_name][best_model_name]

    print(f"\n{feat_set_name}: {best_model_name}")
    print(f"Running {n_bootstrap} bootstrap iterations...")

    bootstrap_r2 = []
    bootstrap_rmse = []

    for i in range(n_bootstrap):
        if i % 100 == 0:
            print(f"  Bootstrap iteration {i}/{n_bootstrap}")

        indices = np.random.choice(len(X_boot), size=len(X_boot), replace=True)
        X_resample = X_boot.iloc[indices].reset_index(drop=True)
        y_resample = y_all[indices]

        pipeline_clone = clone(best_model_pipeline)
        pipeline_clone.fit(X_resample, y_resample)
        y_pred = pipeline_clone.predict(X_resample)

        bootstrap_r2.append(r2_score(y_resample, y_pred))
        bootstrap_rmse.append(np.sqrt(mean_squared_error(y_resample, y_pred)))

    r2_ci = np.percentile(bootstrap_r2, [2.5, 97.5])
    rmse_ci = np.percentile(bootstrap_rmse, [2.5, 97.5])

    print(f"R² Bootstrap 95% CI: [{r2_ci[0]:.4f}, {r2_ci[1]:.4f}]")
    print(f"RMSE Bootstrap 95% CI: [{rmse_ci[0]:.3f}, {rmse_ci[1]:.3f}]")

    fig, axes = plt.subplots(1, 2, figsize=(14, 5))

    ax = axes[0]
    ax.hist(bootstrap_r2, bins=50, alpha=0.7, edgecolor='black')
    ax.axvline(np.mean(bootstrap_r2), color='red', linestyle='--', linewidth=2, label='Mean')
    ax.axvline(r2_ci[0], color='green', linestyle=':', linewidth=2, label='95% CI')
    ax.axvline(r2_ci[1], color='green', linestyle=':', linewidth=2)
    ax.set_xlabel('R²', fontsize=11, fontweight='bold')
    ax.set_ylabel('Frequency', fontsize=11, fontweight='bold')
    ax.set_title(f'Bootstrap Distribution of R²\n{feat_set_name}: {best_model_name}',
                 fontsize=12, fontweight='bold')
    ax.legend()
    ax.grid(axis='y', alpha=0.3)
```

```
    ax = axes[1]
    ax.hist(bootstrap_rmse, bins=50, alpha=0.7, edgecolor='black', color='coral')
    ax.axvline(np.mean(bootstrap_rmse), color='red', linestyle='--', linewidth=2, label='Mean')
    ax.axvline(rmse_ci[0], color='green', linestyle=':', linewidth=2, label='95% CI')
    ax.axvline(rmse_ci[1], color='green', linestyle=':', linewidth=2)
    ax.set_xlabel('RMSE (%)', fontsize=11, fontweight='bold')
    ax.set_ylabel('Frequency', fontsize=11, fontweight='bold')
    ax.set_title(f'Bootstrap Distribution of RMSE\n{feat_set_name}: {best_model_name}',
                fontsize=12, fontweight='bold')
    ax.legend()
    ax.grid(axis='y', alpha=0.3)

    plt.tight_layout()
    plt.savefig(OUTDIR / f"fig{fig_num:02d}_bootstrap_ci.png", dpi=300, bbox_inches='tight')
    plt.close()
    print(f"Figure {fig_num}: Bootstrap confidence intervals")
    fig_num += 1

print("\n" + "="*80)
print("EXHAUSTIVE ANALYSIS COMPLETE")
print("="*80)

# ============================================================================
# SECTION 3.7: STATISTICAL SUMMARY TABLES
# ============================================================================

print("\nCreating statistical summary tables...")

summary_stats = []

for result in all_results:
    residuals = result['residuals']

    summary_stats.append({
        'Model': result['model'],
        'Feature_Set': result['feature_set'],
        'R2_mean': result['test_r2_mean'],
        'R2_std': result['test_r2_std'],
        'RMSE_mean': result['test_rmse_mean'],
        'RMSE_std': result['test_rmse_std'],
        'MAE_mean': result['test_mae_mean'],
        'MAE_std': result['test_mae_std'],
        'MAPE': result['mape'],
        'Max_Error': result['max_error'],
        'Residual_Mean': np.mean(residuals),
        'Residual_Std': np.std(residuals),
        'Residual_Skew': stats.skew(residuals),
        'Residual_Kurtosis': stats.kurtosis(residuals),
        'Shapiro_W': stats.shapiro(residuals)[0],
        'Shapiro_p': stats.shapiro(residuals)[1]
    })

summary_df = pd.DataFrame(summary_stats)
summary_df.to_csv(OUTDIR / "comprehensive_model_statistics.csv", index=False)

# Statistical summary visualization
```

```
fig, axes = plt.subplots(2, 2, figsize=(16, 12))

x_pos = np.arange(len(summary_df))
colors_feat = ['#1f77b4' if '6-feature' in fs else '#ff7f0e' if '5-feature' in fs else '#2ca02c'
               for fs in summary_df['Feature_Set']]

# R² with error bars
ax = axes[0, 0]
ax.barh(x_pos, summary_df['R2_mean'], xerr=summary_df['R2_std'],
       color=colors_feat, alpha=0.7, capsize=3)
ax.set_yticks(x_pos)
ax.set_yticklabels([f"{m}\n{fs}" for m, fs in zip(summary_df['Model'], summary_df['Feature_Set'][illegible]
                   fontsize=8)
ax.set_xlabel('R² (mean ± std)', fontsize=11, fontweight='bold')
ax.set_title('Model Performance with Uncertainty', fontsize=12, fontweight='bold')
ax.grid(axis='x', alpha=0.3)
ax.axvline(summary_df['R2_mean'].max(), color='red', linestyle='--', linewidth=1, alpha=0.5)

# Residual normality (Shapiro-Wilk p-value)
ax = axes[0, 1]
colors_norm = ['green' if p > 0.05 else 'red' for p in summary_df['Shapiro_p']]
ax.barh(x_pos, summary_df['Shapiro_p'], color=colors_norm, alpha=0.7)
ax.axvline(0.05, color='black', linestyle='--', linewidth=2, label='p=0.05 threshold')
ax.set_yticks(x_pos)
ax.set_yticklabels([f"{m}\n{fs}" for m, fs in zip(summary_df['Model'], summary_df['Feature_Set'][illegible]
                   fontsize=8)
ax.set_xlabel('Shapiro-Wilk p-value', fontsize=11, fontweight='bold')
ax.set_title('Residual Normality Test\n(Green: Normal, p>0.05)', fontsize=12, fontweight='bold')
ax.legend()
ax.grid(axis='x', alpha=0.3)

# MAPE comparison
ax = axes[1, 0]
ax.barh(x_pos, summary_df['MAPE'], color=colors_feat, alpha=0.7)
ax.set_yticks(x_pos)
ax.set_yticklabels([f"{m}\n{fs}" for m, fs in zip(summary_df['Model'], summary_df['Feature_Set'][illegible]
                   fontsize=8)
ax.set_xlabel('MAPE (%)', fontsize=11, fontweight='bold')
ax.set_title('Mean Absolute Percentage Error\n(Lower is better)', fontsize=12, fontweight='bold'[illegible]
ax.grid(axis='x', alpha=0.3)

# Max error comparison
ax = axes[1, 1]
ax.barh(x_pos, summary_df['Max_Error'], color=colors_feat, alpha=0.7)
ax.set_yticks(x_pos)
ax.set_yticklabels([f"{m}\n{fs}" for m, fs in zip(summary_df['Model'], summary_df['Feature_Set'][illegible]
                   fontsize=8)
ax.set_xlabel('Maximum Absolute Error (%)', fontsize=11, fontweight='bold')
ax.set_title('Worst-Case Prediction Error\n(Lower is better)', fontsize=12, fontweight='bold')
ax.grid(axis='x', alpha=0.3)

plt.tight_layout()
plt.savefig(OUTDIR / f"fig{fig_num:02d}_statistical_summary.png", dpi=300, bbox_inches='tight')
plt.close()
print(f"Figure {fig_num}: Statistical summary")
fig_num += 1
```

```
# ============================================================================
# FINAL SUMMARY
# ============================================================================

print("\n" + "="*80)
print("ANALYSIS COMPLETE")
print("="*80)
print(f"\nGenerated {fig_num - 1} detailed figures")
print(f"All outputs saved to: {OUTDIR.resolve()}")

best_idx = results_df['test_r2_mean'].idxmax()
best_result = results_df.loc[best_idx]

print(f"\nBEST MODEL:")
print(f"   {best_result['model']} with {best_result['feature_set']}")
print(f"   R² = {best_result['test_r2_mean']:.4f} ± {best_result['test_r2_std']:.4f}")
print(f"   RMSE = {best_result['test_rmse_mean']:.3f} ± {best_result['test_rmse_std']:.3f}%")
print(f"   MAE = {best_result['test_mae_mean']:.3f} ± {best_result['test_mae_std']:.3f}%")

summary_text = f"""

## Dataset
- Samples: {len(y_all)}
- Features: 6 numeric features only
- Target: Solvent retention (%)
- Cross-validation: {N_FOLDS}-fold Stratified at {STRATIFY_THRESHOLD}%

## Models Evaluated
- 6 model types × 3 feature sets = 18 total models
- Extensive hyperparameter tuning via GridSearchCV

## Best Overall Model
- Model: {best_result['model']}
- Feature Set: {best_result['feature_set']}
- Test R²: {best_result['test_r2_mean']:.4f} ± {best_result['test_r2_std']:.4f}
- Test RMSE: {best_result['test_rmse_mean']:.3f} ± {best_result['test_rmse_std']:.3f}%
- Test MAE: {best_result['test_mae_mean']:.3f} ± {best_result['test_mae_std']:.3f}%

## Figures Generated: {fig_num - 1}

### Correlation Analysis
- Pearson vs Spearman comparison
- Individual variable linear analysis
- Correlation matrices (Pearson & Spearman)

### Model Performance
- R² heatmap comparison (all models × feature sets)
- Side-by-side metrics comparison
- Individual model performance plots
- Train vs test R² (overfitting detection)

### Parity Plots
- Best models for each feature set (3 plots)
- All 18 model-feature combinations
```

```
### Residual Analysis
- Complete analysis for best models (3 feature sets × 4 panels)
- Residual plots for all 18 combinations
- Residual standard deviation comparison
- Normality tests

### SVR-Specific
- AIC/BIC analysis and tradeoffs
- Hyperparameter tuning heatmaps (3 feature sets × 3 panels)
- Support vector analysis

### Statistical Summaries
- Comprehensive statistics table
- Error metrics comparison
- Normality tests visualization

## Key Files
- comprehensive_model_statistics.csv - Complete statistics
- all_model_results_detailed.csv - All model results
- svr_information_criteria.csv - SVR AIC/BIC
- correlations_detailed.csv - Correlation analysis

## Total Figures: {fig_num - 1}
All figures saved as high-resolution PNG (300 DPI)
"""

with open(OUTDIR / "ULTRA_COMPREHENSIVE_SUMMARY.md", 'w') as f:
    f.write(summary_text)

print(f"\nSummary report saved to: ULTRA_COMPREHENSIVE_SUMMARY.md")
print(f"\nReview all {fig_num - 1} figures in: {OUTDIR}")

# ==============================================================================
# FULL LEAVE-ONE-OUT R² DROP ANALYSIS (6→5, 5→4, 4→3)
# ==============================================================================

print("\n" + "="*80)
print("FULL LEAVE-ONE-OUT R² DROP ANALYSIS (6→5, 5→4, 4→3)")
print("="*80)

def compute_r2_drop(feature_list, actual_feature_list, baseline_r2):
    """Compute R² after removing each feature from the set."""
    results = []

    for feat_to_remove in feature_list:
        remaining = [f for f in feature_list if f != feat_to_remove]
        actual_remaining = [feature_mapping_rev[f] for f in remaining]

        r2_val = evaluate_feature_set_quick(
            remaining, actual_remaining, data, y_all, cv, y_stratify
        )

        results.append({
            "removed_feature": feat_to_remove,
            "remaining_features": remaining,
            "r2_mean": r2_val,
```

```
            "r2_drop": baseline_r2 - r2_val
        })

    df = pd.DataFrame(results).sort_values("r2_drop", ascending=False)
    df.reset_index(drop=True, inplace=True)
    return df

# Compute baseline R² for full 6-feature model
baseline_r2_6 = evaluate_feature_set_quick(
    six_features,
    actual_six_features,
    data,
    y_all,
    cv,
    y_stratify
)

print(f"\n6-feature baseline R² = {baseline_r2_6:.4f}")

# 6-feature to 5-feature drop
print("\nSTEP 1: 6-FEATURE TO 5-FEATURE R² DROP")

six_feat = six_features
actual_six_feat = actual_six_features
baseline_6 = baseline_r2_6

df_drop_6 = compute_r2_drop(six_feat, actual_six_feat, baseline_6)
df_drop_6.to_csv(OUTDIR / "r2_drop_6_to_5.csv", index=False)
print(df_drop_6)

# 5-feature to 4-feature drop
print("\nSTEP 2: 5-FEATURE TO 4-FEATURE R² DROP")

five_feat = five_features
actual_five_feat = actual_five_features
baseline_5 = best_r2_5

df_drop_5 = compute_r2_drop(five_feat, actual_five_feat, baseline_5)
df_drop_5.to_csv(OUTDIR / "r2_drop_5_to_4.csv", index=False)
print(df_drop_5)

# 4-feature to 3-feature drop
print("\nSTEP 3: 4-FEATURE TO 3-FEATURE R² DROP")

four_feat = four_features
actual_four_feat = actual_four_features
baseline_4 = best_r2_4

df_drop_4 = compute_r2_drop(four_feat, actual_four_feat, baseline_4)
df_drop_4.to_csv(OUTDIR / "r2_drop_4_to_3.csv", index=False)
print(df_drop_4)

# Visualization of R² drops
def plot_r2_drop(df, title, filename):
    fig, ax = plt.subplots(figsize=(8, 5))
    ax.barh(df["removed_feature"], df["r2_drop"], color="tomato", alpha=0.7)
```

```
    ax.set_xlabel("R² Drop", fontsize=12, fontweight="bold")
    ax.set_title(title, fontsize=14, fontweight="bold")
    ax.grid(axis="x", alpha=0.3)
    plt.tight_layout()
    plt.savefig(OUTDIR / filename, dpi=300)
    plt.close()

plot_r2_drop(df_drop_6,
             "6 to 5 Feature Drop (Remove 1 Feature from 6)",
             "fig_r2_drop_6_to_5.png")

plot_r2_drop(df_drop_5,
             "5 to 4 Feature Drop (Remove 1 Feature from Best 5-feature Set)",
             "fig_r2_drop_5_to_4.png")

plot_r2_drop(df_drop_4,
             "4 to 3 Feature Drop (Remove 1 Feature from Best 4-feature Set)",
             "fig_r2_drop_4_to_3.png")

print("\nR² drop analysis (6 to 5, 5 to 4, 4 to 3) completed and saved.")

# ============================================================================
# EXPORT SVR PARITY DATA FOR 6/5/4 FEATURE SETS (POLYMER-SEPARATED CSV)
# ============================================================================

print("\n" + "="*80)
print("EXPORTING SVR PARITY CSV (POLYMER SEPARATED)")
print("="*80)

for feat_set in ['6-feature', '5-feature', '4-feature']:

    svr_result = [
        r for r in all_results
        if r['model'] == 'SVR' and r['feature_set'] == feat_set
    ][0]

    y_true = svr_result['predictions'] * 0 + y_all
    y_pred = svr_result['predictions']
    residuals = y_true - y_pred
    polymer_list = data['Polymer'].values

    df_out = pd.DataFrame({
        'Polymer': polymer_list,
        'True': y_true,
        'Pred': y_pred,
        'Residual': residuals
    })

    save_name = f"svr_parity_{feat_set.replace('-', '_')}.csv"
    df_out.to_csv(OUTDIR / save_name, index=False, encoding='utf-8-sig')

    print(f"Saved: {save_name}")
```
